# Attachment site recognition and regulation of directionality by the serine integrases

**DOI:** 10.1093/nar/gkt580

**Published:** 2013-07-02

**Authors:** Karen Rutherford, Peng Yuan, Kay Perry, Robert Sharp, Gregory D. Van Duyne

**Affiliations:** ^1^Department of Biochemistry and Biophysics, Perelman School of Medicine, University of Pennsylvania, Philadelphia, PA 19104, USA and ^2^NE-CAT and Department of Chemistry and Chemical Biology, Cornell University, Building 436E, Argonne National Laboratory, 9700 S. Cass Avenue, Argonne, IL 60439, USA

## Abstract

Serine integrases catalyze the integration of bacteriophage DNA into a host genome by site-specific recombination between ‘attachment sites’ in the phage (*attP*) and the host (*attB*). The reaction is highly directional; the reverse excision reaction between the product *attL* and *attR* sites does not occur in the absence of a phage-encoded factor, nor does recombination occur between other pairings of attachment sites. A mechanistic understanding of how these enzymes achieve site-selectivity and directionality has been limited by a lack of structural models. Here, we report the structure of the C-terminal domains of a serine integrase bound to an *attP* DNA half-site. The structure leads directly to models for understanding how the integrase-bound *attP* and *attB* sites differ, why these enzymes preferentially form *attP* × *attB* synaptic complexes to initiate recombination, and how *attL* × *attR* recombination is prevented. In these models, different domain organizations on *attP* vs. *attB* half-sites allow attachment-site specific interactions to form between integrase subunits via an unusual protruding coiled-coil motif. These interactions are used to preferentially synapse integrase-bound *attP* and *attB* and inhibit synapsis of integrase-bound *attL* and *attR*. The results provide a structural framework for understanding, testing and engineering serine integrase function.

## INTRODUCTION

**Figure 1.**
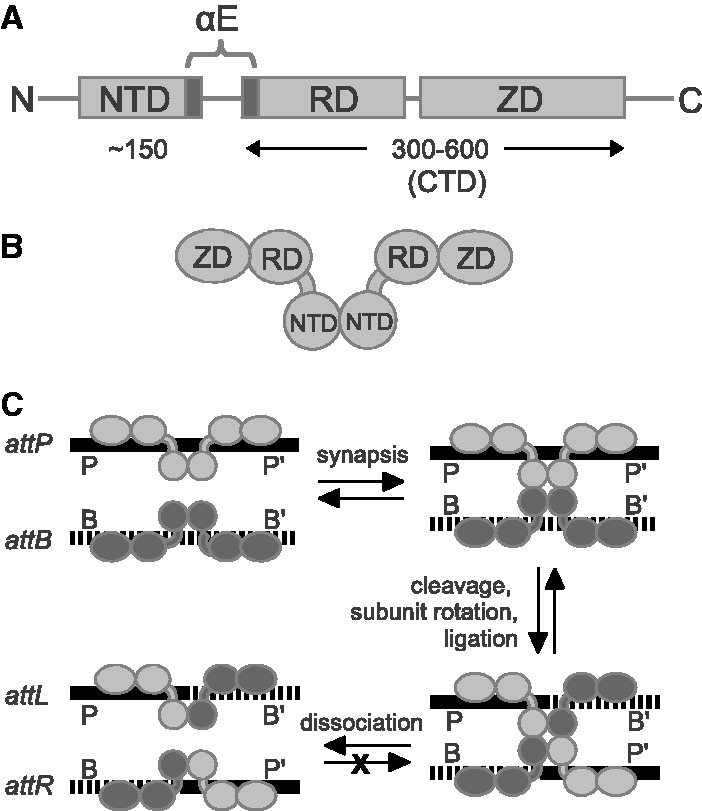
LSRs. (A) LSR domain organization. An NTD is conserved with the resolvases/invertases. The LSR CTD varies in size and contains a RD and ZD. A long helix (αE) is shared between the NTD and RD. (B) Schematic of a LSR dimer in the DNA-bound form; dimerization is mediated by the catalytic domains and αE. (C) Schematic of the highly directional integration reaction mediated by LSRs. The int-bound *attL* and *attR* sites are blocked from entering the reverse pathway to generate *attP* and *attB* sites. The *attP* and *attB* half-sites are referred to as P/P′ and B/B′, respectively.

The large serine recombinases (LSRs) are DNA-rearranging enzymes that are members of the serine recombinase superfamily (1,2). Many LSRs are bacteriophage integrases (often referred to as serine integrases) whose function is to integrate the phage genome into a host chromosome by using short specific DNA sequences, or ‘attachment sites’, in the virus (*attP*) and in the host (*attB*). During the integration reaction, LSRs bind to *attP* and *attB* as dimers, mediate association of the sites to form a tetrameric synaptic complex and catalyze strand exchange to generate an integrated prophage and new attachment sites, *attL* and *attR* (Figure 1). The reverse reaction, where the prophage is excised by site-specific recombination between *attL* and *attR* sequences, does not occur in the absence of a phage-encoded recombination directionality factor (RDF). Additional functions for LSRs include movement of bacterial resistance genes via transposition (3,4) and via other mobile elements (5), and developmentally programmed DNA rearrangements (6).

The simplicity and highly directional nature of the integration reaction catalyzed by serine integrases has led to their use as powerful genome engineering tools (1,7). There are no requirements for accessory proteins, auxiliary DNA sequences, specific DNA topologies or supercoiling, and the short (40–50 bp) attachment sites allow for simple manipulation of substrates. Because of these properties, the *attP* × *attB* integration reaction can be performed with high specificity and directionality in inter- and intramolecular contexts, and in a variety of eukaryotic cells (8–12). The bacteriophage φC31 integrase has also shown promise for use in gene therapy applications (13).

The LSRs share a catalytic domain with the smaller resolvase/invertase enzymes (Figure 1A), and there is a strong consensus that the LSRs use the same mechanism of strand exchange (2,14). However, the resolvase/invertase enzymes function under strict topological contexts and do not share the large C-terminal domain (CTD) that is responsible for coordination of unique LSR activities (15). Work in the bacteriophage φC31 and Bxb1 integrase systems has shown that site-selectivity and directionality are regulated at the synapsis step of the recombination pathway (16,17). It has been difficult, however, to understand on a molecular level why *attP* and *attB* sites can be efficiently synapsed and recombined by a serine integrase, while other combinations of sites associate poorly or not at all. The prevailing view has been that the integrases bound to *attP* and *attB* are related by a ‘conformational change’ directed by the different DNA sequences present in the two sites and that these changes are responsible for regulation (16,18).

Our understanding of how serine integrases function on a molecular level has been limited by a lack of structural data for the C-terminal region (∼70%) of the enzyme and consequently the mechanisms of site recognition, site-selectivity and directionality, and facilitation of synapsis have remained a mystery. A particularly puzzling question deals with the nature of *attP* vs. *attB* binding. These sites share only limited sequence similarity in most systems, yet the same LSR dimer binds to both with high affinity. Given that differences between the integrase–*attP* (int–*attP*) and integrase–*attB* (int–*attB*) complexes are key to understanding how the LSR enzymes function, experimental structures of LSR–DNA complexes have been an important goal of this field.

Here we report the structure of a *Listeria* phage integrase CTD bound to its *attP* half-site. The structure reveals that the LSR CTD is composed of a mixed α/β recombinase domain (RD) linked to an unusual zinc ribbon domain (ZD), which together interact with the entire 25 bp of the *attP* half-site through extensive major and minor groove contacts. A coiled-coil (CC) motif is inserted between helices in the ZD and extends away from the protein–DNA complex. The structure leads directly to a model for *attB*-binding that involves a reorganization of ZDs in the context of an otherwise similar core int–DNA complex. The structural data, combined with results of recombination and DNA-binding experiments for integrase and attachment site mutants, lead to an architectural model for the recombination pathway that explains how LSR enzymes regulate site-selectivity and directionality. In this model, the *attP* and *attB* half-sites dictate the spatial organization of ZDs that are available to facililtate synapsis of a given pair of attachment sites. In the *attP* × *attB* synaptic complex, the CC motifs from juxtaposed half-sites can interact, but they are improperly positioned in other site pairings. The *attL* and *attR* sites are unique in that the ZDs are positioned to form an autoinhibited complex in which the CC motifs from the *attP*- and *attB*-derived half-sites can interact with one another and prevent synapsis. The structure-based model also suggests how RDF proteins might function to destabilize this inhibitory complex.

## MATERIALS AND METHODS

### Crystallization and structure determination

LI integrase CTD (133–452) was expressed with a C-terminal hexahistidine tag in BL21(DE3) cells. Selenomethionine-labeled CTD was expressed in a synthetic minimal medium (19). The LI CTD was purified using Ni-NTA, MonoS cation exchange and Superdex-200 size exclusion columns. The DNA half-site used for crystallization was a 25-mer duplex based on the A118 P-arm, with 5′-G and 5′-C overhangs on the top and bottom strands, respectively. Protein–DNA complex crystals were grown by hanging drop vapor diffusion from drops containing 30 μM CTD, 45 μM DNA, 25 mM 4-(2-hydroxyethyl)-1-piperazineethanesulfonic acid (HEPES), pH 7.0, 50 mM NaCl, 13 mM CaCl_2_, 9% 2-methyl-2,4-pentanediol (MPD), 3% glycerol and 0.5 mM tris(2-carboxyethyl)phosphine (TCEP) that were equilibrated against reservoirs containing 50 mM HEPES, pH 7.0, 26 mM CaCl_2_, 17% MPD and 6% glycerol at 21°C. The crystals are cubic, space group I23, with a = 290.8 Å.

Diffraction data were measured at selenium and zinc absorption edges to obtain anomalous scattering data (Table 1). Initial phases based on Se SAS phasing revealed electron density for the DNA and for the integrase domains after solvent-flattening. Noncrystallographic symmetry (NCS) averaging of the four independent CTD–DNA complexes in the asymmetric unit and flattening of the 74% solvent volume resulted in electron density that was readily interpretable everywhere except for the CC motifs. The CC motifs were not subjected to NCS averaging and were identified in difference electron density maps following refinement. The structure was refined to 3.2 Å resolution with NCS restraints applied to the RD, ZD and DNA, with *R*_work_ = 0.24 and *R*_free_ = 0.26 (Table 1). Coordinates and structure factors were deposited with the Protein Data Bank, code 4KIS.

**Table 1.** Crystallographic data

	Native		Native merge	Zn		
Wavelength (λ)	0.97921	0.97921	0.97921	1.28255 (peak)	1.28295 (inflection)	
Resolution (Å)	2.85	3.00	3.20	4.05	4.15	
Completeness (%)	69.5	86.4	98.4 (80.7)	99.8 (99.9)	99.8 (99.9)	
*R*_merge_	0.101	0.112	0.124 (0.79)	0.085 (0.60)	0.087 (0.66)	
*I/σ*	9.8	11.0	15.7 (0.9)	20.2 (2.3)	18.4 (2.1)	
Redundancy	3.1	3.4	6.5 (3.2)	4.3 (4.3)	4.3 (4.3)	
Unique Reflections	65 293	70 069	68 573	32 541	30 304	

Selenomethionine						SeMet merge

Wavelength (λ)	0.97918	0.97918	0.97918	0.97918	0.97918	0.97918
Resolution (Å)	4.10	4.30	4.05	4.30	4.15	4.20
Completeness (%)	99.9 (100)	99.9 (99.7)	100 (100)	99.2 (98.3)	99.9 (100)	100 (100)
*R*_merge_	0.130 (0.98)	0.160 (1.00)	0.14 (0.00)	0.123 (0.85)	0.165 (0.75)	0.201 (1.0)
*I/σ*	16.4 (2.0)	16.4 (2.2)	25.0 (2.5)	17.0 (2.3)	12.3 (2.5)	32.9 (5.8)
Redundancy	7.4 (7.5)	8.9 (7.7)	17.2 (17.1)	7.3 (7.2)	7.2 (6.7)	47.6 (41.5)
Unique reflections	34 004	33 934	33 692	28 852	30 733	29 079
SAD phasing (SOLVE/RESOLVE)						
Bayesian CC score	44.6					
Resolution (Å)	5.3					
Overall FOM	0.46					
Refinement
*R*_work_	0.236					
*R*_free_^a^	0.256					
Number of atoms						
Protein	10 086					
DNA	4240					
Zinc Ions	6					
Solvent	8					
RMSD						
Bond Lengths (Å)	0.009					
Bond Angles (°)	1.336					

*R*_merge_ = Σ|I_h_ − <I_h_>|Σ I_h_, where <I_h_> is the average intensity over symmetry equivalent measurements; R-factor, Σ |Fobs – Fcalc| / Σ Fobs, where summation is data used in refinement. Numbers in parentheses represent values in the highest-resolution shell.

^a^*R*_free_ was calculated with 5% of the data.

### DNA-binding assays

DNA probes for electrophoretic mobility shift assays (EMSA) were ^32^P-labeled 119-bp duplexes containing 56-bp LI *attP* or *attB* sites. Binding reactions contained 20 mM Tris–HCl, pH 7.4, 150 mM KCl, 2 mM dithiothreitol (DTT), 25 µg/ml bovine serum albumin (BSA), 25 µg/ml salmon sperm DNA, 5% glycerol and ∼1 nM DNA, and were incubated for 30 min at 30° before electrophoresis on 6% polyacrylamide (37.5:1) using Tris-glycine buffer, pH 8.3, at 15°C.

### Recombination assays

Intramolecular recombination in *Escherichia coli* was tested using the F′-reporter previously described (20), where excision of a transcriptional terminator flanked by attachment sites leads to streptomycin resistance and a *lac+* phenotype. Activity was defined as the fraction of colonies that are streptomycin resistant following transformation of an integrase expression plasmid. Intermolecular recombination in *E. coli* was measured by transformation of a suicide R6kγ plasmid containing a single attachment site into strain CSH142 containing a partner attachment site in single copy on an F′-episome and an integrase expression plasmid. Because the R6kγ plasmid cannot replicate in this strain, ampicillin-resistant colonies are only observed when the plasmid integrates into the F′. Activity was defined as the number of ampicillin-resistant colonies obtained relative to wild-type (WT) *attP* integrating into *attB*. All assays were performed in triplicate.

### Models of synaptic complexes

The full-length int–*attP* complex model was constructed by superposing base pairs 1–5 onto the corresponding base pairs in each half-site of the γδ-resolvase/*res* site I complex (pdb 1GDT) and introduction of phosphodiester linkages at the crossover site. The model contains a symmetric *attP* site composed of two P arms, LI integrase CTD (133–452) and γδ-resolvase (1–123). To generate the *attB* complex model, the ZDs of the *attP* complex were shifted 5 bp toward the crossover site, using a transformation derived from superposition of DNA half-site base pairs 16–24 onto base pairs 11–19. Synaptic complex models were generated by superposition of LI integrase–CTD half-site complexes onto the DNA duplex segments of a tetrameric γδ-resovase/DNA complex (pdb 1ZR4).

Structure illustrations were produced using UCSF Chimera (21) and Pymol (DeLano Scientific, 2002). Additional experimental methods details can be found in the Supplementary Text.

## RESULTS

### Crystallization and structure determination of the LI integrase CTD–DNA complex

**Figure 2.**
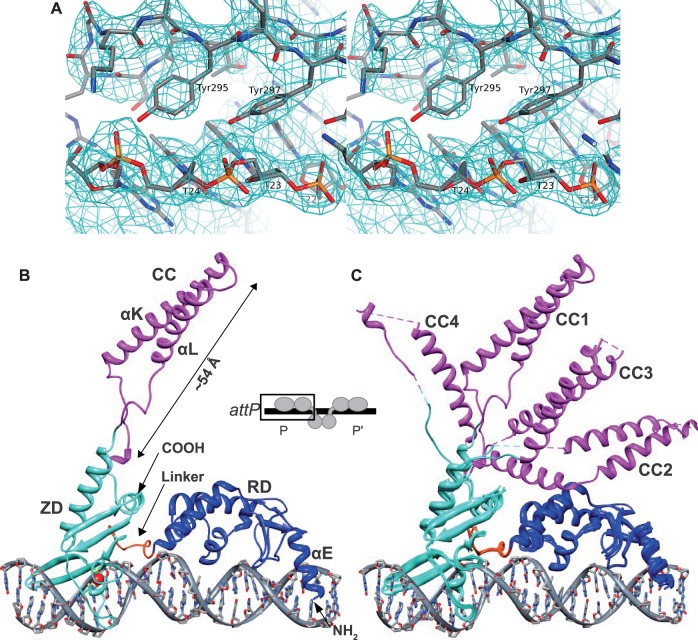
Structure of LI integrase CTD bound to the A118 *attP* P-arm. (**A**) Electron density at the int–DNA interface showing a key major groove interaction where the side chains of Tyr295 and Tyr297 contact the 5-methyl groups of T22, T23 and T24. The density shown is from a weighted 2F_o_-F_c_ map at 3.2 Å, contoured at 1.4σ. (**B**) Overall structure of the complex. The inset shows a schematic of the structure in the context of the full int–*attP* complex. The minor groove binding RD–ZD linker is indicated and the coordinated zinc ion is drawn as a red sphere. The half-site DNA contains 25 bp of the *attP* P-arm, including the first G of the crossover site. (**C**) Superposition of the four complexes in the asymmetric unit by structural alignment of the RD (Cα root-mean square deviation (RMSD) = 0.42 Å), ZD (Cα RMSD = 0.66 Å) and DNA (C1′ RMSD = 0.86 Å). The four CC motifs adopt different orientations.

We examined several serine integrase systems to identify one that was both biochemically tractable and showed promise for structural investigations of int–att site complexes. The integrase from a *Listeria innocua* prophage (GenBank CAC97653), referred to here as LI integrase, was particularly promising based on initial experiments and became the model system for our structural and biochemical studies. LI integrase differs by 11 conservative amino acid changes (98% identical) from the bacteriophage A118 integrase (22) and has similar attachment site sequences (Supplementary Figure S1). We crystallized the LI integrase CTD (residues 133–452) bound to a 26-bp A118 *attP* half-site and determined the structure using anomalous scattering data from selenomethionine-substituted protein and intrinsically bound Zn ions. Although the resolution of the crystals was limited to 3.2 Å, a high solvent content of 74% increased the number of experimental data to that of a 2.5 Å structure with 50% solvent, and NCS averaging of four independent complexes in the asymmetric unit led to strong phase improvement and high-quality electron density maps. Crystallographic and refinement data are shown in Table 1, and electron density at the int–DNA interface is shown in Figure 2A.

### Conserved structure of the LSR CTD

The integrase CTD is composed of two structural domains: a mixed α/β ‘recombinase domain’ (RD) and a zinc-nucleated domain (ZD) containing an embedded CC motif that extends away from the RD and the DNA (Figure 2B). The RD and ZD are connected by an 8-residue linker. The construct used for crystallization contains part of the helix (αE) that connects the N-terminal catalytic domain (NTD) and RD and is involved in formation of the subunit rotation interface during strand exchange (23). Together with αE, residues in the integrase CTD make contiguous contact with the entire *attP* half-site via alternating major and minor groove interactions (Figure 2B). The full-length integrase dimer is therefore expected to contact 50 bp of the *attP* site. The four independent complexes in the asymmetric unit of the crystal are similar everywhere except in the CC motifs, which adopt distinct trajectories (Figure 2C).

**Figure 3.**
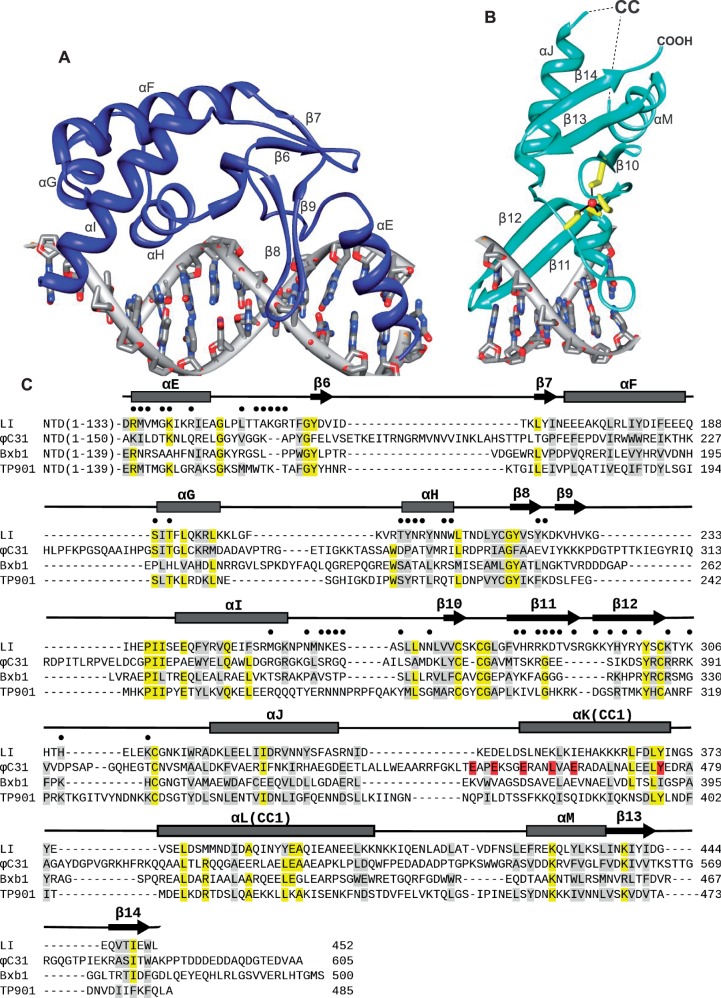
Conserved LSR domains. (**A**) Structure of the RD. Helices αF, αG and αH form a HTH subdomain that is similar to that found in the resolvase/invertase enzymes (see also Supplementary Figure S2). (**B**) Structure of the ZD. Zinc is drawn as a red sphere and the four coordinating cysteine residues are drawn as sticks. (**C**) Sequence alignment of the CTDs for four serine integrases. Secondary structure assignments for LI integrase are indicated. Residues that are identical in 3–4 of the four sequences are highlighted in yellow and similar residues in gray. Residues that contact the *attP* site are indicated by black circles. Hyperactive and defective φC31 mutants in the CC motif are indicated in red. The alignment was generated using Clustal W (24) and was then adjusted manually based on the LI integrase CTD structure and secondary structure predictions. See Supplementary Figure S1 for full-length alignments of A118-like integrases.

The RD is moderately well-conserved among the LSRs (2). Briefly, this domain consists of the C-terminal end of αE and a four-stranded β-sheet embedded in a core 4-helix bundle (Figure 3A). Although there is little sequence similarity between the LSR RD and the smaller helix-turn-helix (HTH) domain that constitutes nearly the entire CTD in the related resolvase/invertase enzymes (15), a structurally similar motif is nonetheless found at the core of the LSR RD (Supplementary Figure S2). A short stretch of amino acids that links αE with β6 and tracks along the minor groove is also structurally similar to that seen in the resolvases. Indeed, αE and the αE-β6 linker form similar interactions with the *attP* site as observed for the corresponding regions of the γδ-resolvase/DNA complex (25). The αE-β6 linker plays an important functional role because mutations in this region of the integrase or the attachment site can have a profound effect on activity (16,26).

The presence of a zinc-coordinating domain was anticipated from LSR sequence analyses and experiments confirming that zinc is required for bacteriophage φC31 integrase activity (27). Our structure indicates that this domain is a member of the zinc ribbon class, where zinc is typically coordinated by four cysteine residues located within two β-hairpins (28). For LI integrase, zinc is coordinated by Cys274 and Cys277, positioned in the β10–β11 turn, and Cys302 and Cys314 positioned in the loop between β12 and αJ (Figure 3B and C). The zinc-coordinating strands form a continuous β-sheet, with β11–β12 engaging the DNA major groove. The β12–αJ turn contains a short 3_10_-helix that extends the major groove interaction to cover 9 bp of the *attP* half-site. The complete ZD consists of the zinc-nucleated β-structure described above, followed by helical segments that precede (αJ) and follow (αM) the CC insertion, and a final β-hairpin (β13–β14) that extends the β-sheet. The αM–β13 region corresponds to a previously identified motif that is rich in Val, Leu and Ile (2). There are no matches to this domain in the Dali database (29), suggesting that this is the first structural example for this zinc-nucleated DNA-binding module class.

The CC motif is the most conspicuous structural element in the serine integrase CTD, extending away from the ZD and the protein–DNA interface (Figure 2B and C). This motif consists of two antiparallel helices (αK and αL) connected to αJ and αM of the ZD by flexible linkers. The helices are connected by a turn that forms the tip of the CC and includes several hydrophobic residues (I370, Y374, V376; Supplementary Figure S3). The amount of observed helical structure varies among the four independent CC motifs, but it appears that both αK and αL may be extended by 1–2 helical turns at their N- and C-terminal ends, respectively, relative to the assignment shown in Figure 3C. Although electron density for the CC motifs is weak, two methionine residues (M382 and M383) in αL facilitated the sequence assignment because their locations could be identified from selenomethionine anomalous scattering measurements. Electron density for one of the CC motif helical regions is shown in Supplementary Figure S3.

The lengths of αK and αL are predicted to be similar among different LSRs (Figure 3C), but the size of the linker regions at the base of the CC varies. These segments are not well ordered in the LI integrase CTD–DNA complex structure and adopt distinct conformations in each of the four independent molecules. The linker regions of some serine integrases also contain several proline residues (e.g. φC31 integrase; Figure 3C), suggesting that a degree of flexibility in CC trajectory is conserved. The best-defined CC motif in our structure is CC1, which spans 54 Å as measured from Ala338 at the end of αJ to Tyr374 at the center of the turn between αK and αL (Figure 2B). We observed some lattice contacts between CC motifs that could be related to the types of interactions made during recombination, but did not test these experimentally in the current study.

### Recognition of the *attP* sequence by A118 family integrases

**Figure 4.**
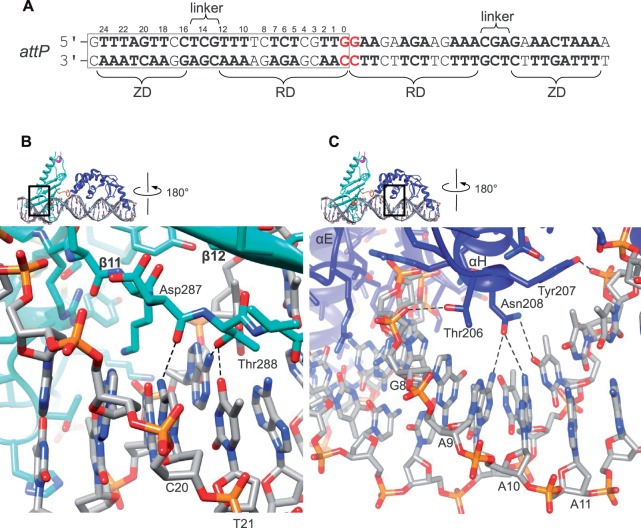
Recognition of the *attP* site by LI integrase. (**A**) Sequence of the A118 *attP* site. The regions contacted by the RD, the ZD and the RD–ZD linker are indicated and the P-arm sequence contained in the crystal structure is boxed. Residues identical in the two half-sites are in bold and the central dinucleotide is colored red. (**B**) Close-up of the ZD–*attP* interface illustrating the interactions between Asp287 and Thr288 in the β11–β12 hairpin and the C20 and T21 base pairs. (**C**) Close-up of the RD-major groove interface where αH interacts. Tyr207 interacts with the 5-methyl groups of T10 and T11 and Asn208 forms a network of hydrogen bonds to the A-T base pairs at positions 9–11.

The A118 *attP* site is pseudo-palindromic, with similar half-site sequences (Figure 4A). The integrase CTD makes numerous interactions with the bases in the major and minor grooves and with the sugar–phosphate backbone throughout the 25-bp half-site. A schematic of all observed contacts is provided in Supplementary Figure S4 and residues that contact DNA are indicated in Figure 3C. Throughout the int–DNA interface, solvent-mediated interactions are likely to contribute to site recognition. Because the resolution of our structural model does not allow clear visualization of this level of detail, we restrict our discussion here to those recognition elements that are clearly observed in all four independent molecules and do not involve bound solvent.

The ZD recognizes a continuous 9-bp ‘ZD motif’ at the distal end of the half-site (positions 16–24 in Figure 4A). At the end of the ZD motif, the side chains of Tyr295 and Tyr297 from β12 lie on the surface created by the 5-methyl groups of T24, T23 and T22 (Figure 2A). This interaction resembles that used by the *vaccina* virus topoisomerase in which tyrosine side chains from a β-hairpin contact three consecutive cytidine residues in its recognition sequence (30). In the β11 strand, the backbone carbonyl of Asp287 forms a hydrogen bond with the 4-amino group of C20 and Thr288 hydrogen bonds to T21 (Figure 4B). Residues in this region of φC31 integrase have been shown to be important for DNA-binding (27), supporting the idea that a similar β-hairpin-DNA interface is formed in all LSRs. A recent study of the Bxb1 integrase *attP* site also indicates that the corresponding flanking sequences are important for integrase binding and recombination (31). Additional direct interactions in the ZD-binding region are illustrated in Supplementary Figure S4.

Despite its large 13-bp contact area, the RD interacts directly with only 3 bp in the major groove. From αH, Tyr207 contacts the 5-methyl group of T11, and Asn208 forms a network of hydrogen bonds to the A-T base pairs involving T9 and T10 (Figure 4C). The basis for sequence recognition in this region of *attP* is less clear than for the ZD-binding motif because it appears that other sequences at positions 9–11 could also form productive interactions. Presumably, visualization of the role of solvent-mediated interactions will provide additional insights into the RD-major groove interface.

The CTD also forms numerous direct contacts to bases in the minor groove, which contribute to the specificity of int–*attP* recognition. An example of such an interaction involves Asn259, whose side chain hydrogen bonds to N2 and N3 of G14 (Supplementary Figure S4B). This residue is located in the interdomain linker between the RD and ZD. In addition, the Met136 side chain from αE intercalates between the G0 and T1 bases in all four int–DNA complexes in the asymmetric unit (Supplementary Figure S4C). Although the *attP* half-sites are not connected in the crystal lattice to produce intact *attP* sites, they do form continuous B-DNA helices. In each case, the DNA helix accommodates the distortion introduced by Met136 intercalation. A similar interaction was observed in the γδ-resolvase–*res* site I complex where the methyl group of Thr126 intercalates between the corresponding base pairs in one subunit of the dimer–DNA complex (25). This interaction may therefore be a functionally important feature of serine recombinases, given its proximity to both the DNA bend in the resolvase–DNA complex and the scissile phosphate located on the opposite strand.

### Roles of the zinc ribbon and CC domains in att-site binding and recombination

**Figure 5.**
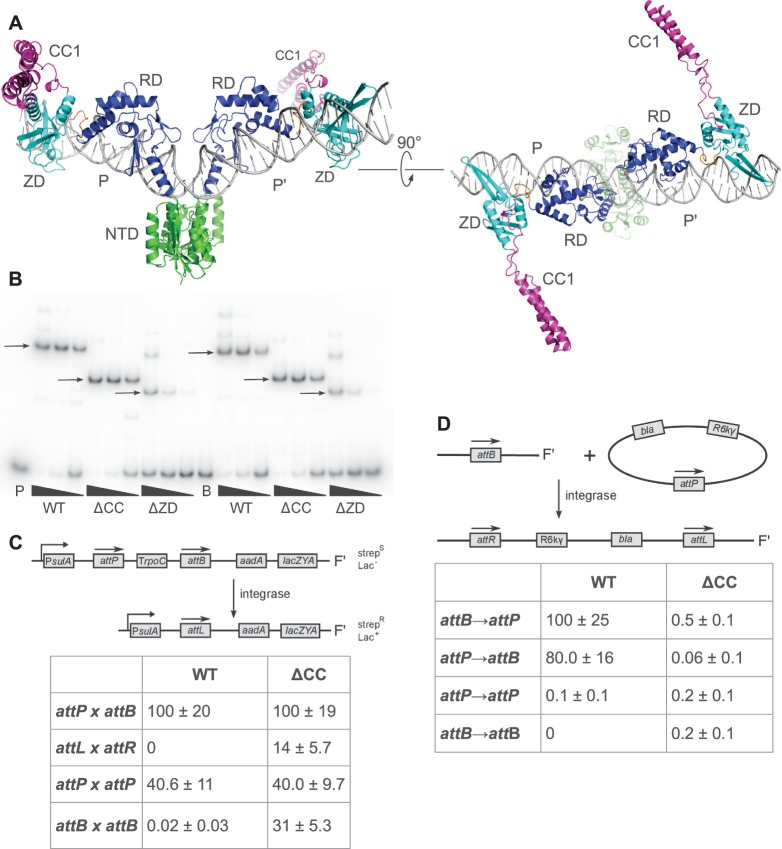
Role of the CC motif in DNA-binding and recombination. (**A**) Model of LI integrase bound to the full-length *attP* site, using the central DNA bend seen in the γδ-resolvase/site I complex (25). (**B**) EMSA of purified WT and mutant LI integrases binding to ^32^P-labeled 119-bp probes containing LI *attP* or *attB* sites. Integrase concentrations were 500, 100 and 20 nM. WT: full length (residues 1–452); ΔCC: Δ(342–416), ΔZD: 1–264. WT and ΔCC integrases bind to *attP* and *attB* with similar affinities. The ΔZD integrase binds *attP*/B with ∼25-fold weaker affinity. (**C**) Intramolecular recombination catalyzed by WT and ΔCC integrases in *E.coli.* Activity is the percent of transformants that are streptomycin resistant. The ΔCC enzyme is promiscuous in this assay. Less than 0.1 % of colonies are streptomycin-resistant when a plasmid expressing the S10A catalytic mutant is transformed. (**D**) Intermolecular recombination catalyzed by WT and ΔCC integrases in *E.coli.* Activity is the number of transformants obtained relative to integration of *attB* plasmid into a single copy *attP* site by WT integrase. Only *attP→attB* and *attB→attP* integration are efficiently catalyzed by the WT integrase, and the ΔCC enzyme shows no activity for any site pairing tested. No colonies are obtained when a suicide plasmid lacking an attachment site is transformed.

To visualize the relative positioning of integrase domains on the full *attP* site, we constructed a model of LI integrase bound to *attP* using the γδ-resolvase/*res* site I complex as a guide (Figure 5A). Given the conserved structures of the NTD and αE in the LSR and resolvase enzymes (32) and the similarities noted for αE, the αE-RD linker and their modes of interaction with DNA, there are few degrees of freedom involved in constructing this model. Indeed, when the innermost 5 bp of the LI CTD–DNA half-site complex are superposed onto the corresponding base pairs of the resolvase–DNA complex, the αE and αE-RD linker segments are well-aligned between LI integrase and γδ-resolvase, suggesting a high degree of similarity in this region at the structural level (Supplementary Figure S2).

In the int–*attP* model, the ZDs, and consequently the CC motifs, are oriented in opposite directions from one another with a ZD–ZD distance of 126 Å between Ala338 at the end of αJ. The model therefore indicates that the CC motifs are unlikely to interact with one another when an integrase dimer is bound to *attP* because the CC motifs (∼54 Å in length) would only be able to span a distance of ∼108 Å (Figure 5A). This conclusion does not depend on details of the *attP* complex model that might differ between int–*attP* and resolvase–*res* complexes, such as small differences in the degree of DNA bending at the center of the site. Whereas there is a clear structural role for the ZD in DNA binding, it does not appear that the CC motif contributes to *attP* recognition or binding affinity.

To test these ideas, we compared the ability of WT LI integrase to bind to *attP* and *attB* and carry out both intra- and intermolecular recombination with that of integrase mutants where either the CC motif (ΔCC) or the entire ZD (ΔZD) has been deleted. As shown in Figure 5B, WT integrase binds to *attP* and *attB* with a K_d_ of ∼20 nM, similar to the affinities reported for A118 integrase (33). The ΔCC integrase binds to both *attP* and *attB* with WT affinity, demonstrating that the CC motif does not contribute substantially to DNA binding and that this motif can be excised from the integrase without affecting its ability to bind attachment sites. As expected, the ΔZD integrase is deficient in DNA binding, consistent with the properties of EDTA-treated integrase and mutants targeting the zinc-coordinating residues in the φC31 system (27), as well as truncations in this region of LSRs (16,27,34).

We next tested the ability of WT and ΔCC integrases to carry out intramolecular recombination in *E.coli* (Figure 5C). The WT integrase efficiently carries out *attP* × *attB* excision, but is inactive for the *attL* × *attR* and *attB* × *attB* reactions. Interestingly, we observed a high level of intramolecular *attP* × *attP* recombination. Recombination between *attP* sites is not observed for most serine integrases (2), but has been reported for the A118 integrase (33) and the CcrA/CcrB recombinases from the SCCmec element (35). Surprisingly, the ΔCC enzyme catalyzes excision for all four site combinations tested. The promiscuous nature of this mutant indicates that the CC motif is not strictly required for recombination, but plays a key role in site-selectivity and directionality.

To compare the ability of WT and ΔCC integrases to carry out recombination under conditions where they must also be capable of efficient synapsis, we carried out integration experiments in *E.coli* (Figure 5D). The results for intermolecular recombination were different than those observed for intramolecular recombination. The WT enzyme integrates an *attP*-containing plasmid into a single-copy *attB* site and integrates an *attB* plasmid into an *attP* site. However, we observed only background levels of activity for *attP* × *attP* and *attB* × *attB* integration by the WT integrase and similarly low activity for all four site combinations tested with the ΔCC integrase. Similar results were obtained using an *in vitro* recombination assay with linearized substrates (Supplementary Figure S5). The simplest interpretation of these results is that the CC motif is required for efficient synapsis, but is only able to facilitate synapsis for the *attP* × *attB* reaction. In the intramolecular reaction (Figure 5C), synapsis is facilitated by both the proximity of sites and by DNA supercoiling, allowing the ΔCC integrase to function as an unregulated resolvase in this context. These results also indicate that the integrase CC motif functions as both a positive and negative regulator because *attL* × *attR* and *attB* × *attB* recombination are efficiently blocked by WT integrase in the intramolecular assay.

### Integrase binding to the *attB* site

**Figure 6.**
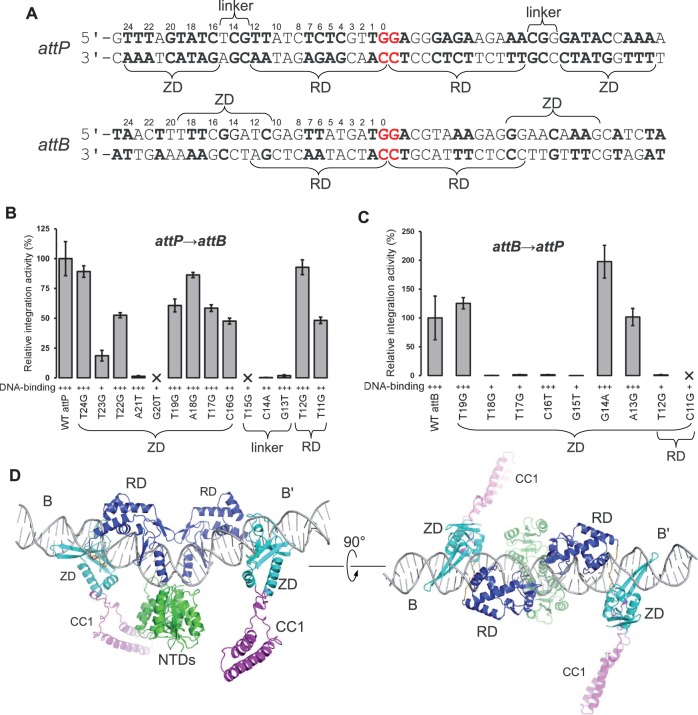
Model of serine integrase–*attB* site binding. (**A**) Comparison of LI *attP* and *attB* sequences. The ZD motif identified in the LI int–DNA structure (5′-TTTAGTATC) is present in *attB*, shifted by 5 bp toward the center of the site relative to *attP*. The RDs are assumed to bind to the same sites in *attP* and *attB*. Residues that are identical in both half-sites are in boldface and the crossover dinucleotide is in red. (**B** and **C**) Integration and DNA-binding activities for *attP* and *attB* mutants, respectively. DNA-binding assays were performed as in Figure 5B and integration assays as in Figure 5D. DNA-binding was scored as triple plus: similar to WT; double plus: slightly defective; plus: severely defective (see also Supplementary Figure S5). (**D**) Model of the int–*attB* complex generated by shifting the ZDs by 5 bp toward the center of the *attP* complex.

To understand how the LSR CC motif functions to regulate recombination, it is essential to understand what is different about how integrase binds to *attP* vs. *attB* sites. In the A118, Bxb1, φRV1 and φBT1 LSR systems, the minimum functional *attB* sites are ∼10 bp shorter than the minimal *attP* sites (16,33,36,37). For φC31 integrase, the difference between minimal sites was originally reported as 5 bp (8). However, the LI int–DNA structure suggests that φC31 integrase contacts a larger region of its *attP* site than what is implied by the minimum functional sequence as measured in the context of an intact plasmid. These observations, combined with identification of the ZD motif shown in Figure 4A and the modular nature of the ZD, led us to test the following hypothesis: ZD motifs can be readily identified in the LI *attB* site sequence, shifted 5 bp toward the central dinucleotide (Figure 6A). If we assume that the RD binds to the *attB* site in a similar manner to that observed in the int–*attP* structure, then the ZD could be readily accommodated in the major groove adjacent to the RD’s HTH motif. The ZD would therefore be located 1/2 of a helical turn away from its position in the *attP* half-site, resulting in a different orientation and potential range of CC motif trajectories.

To test this *attB* binding model, we made a series of substitutions in the LI *attP* and *attB* sites and measured the ability of LI integrase to bind to and carry out integration with these sites. In each case, the modified sites contained two point substitutions corresponding to symmetric replacements in both half-sites. Our choice of substitutions was based on changing the target base to a residue that was not present in either half-site and that was predicted to cause disruption to the int–DNA interface based on the CTD–*attP* structure. The results for *attP* are largely consistent with the observed LI int–DNA interface (Figure 6B). Disruption of the central thymidine (T23) of the thymidine–tyrosine cluster (Figure 2A) and of the T21–Thr288 and C20–D287 contacts in the zinc motif (Figure 4B) have strong effects on integration, as do substitutions that modify the minor groove where the RD–ZD linker is engaged (G13–T15). Interactions involving the HTH motif (T11–T12) are more tolerant to substitutions. In most cases, reduction of integration activity is accompanied by reductions in DNA-binding affinity. However, substitutions at T21 and G13 of *attP* result in sharp reductions in integration activity, but retain high integrase binding affinities.

The *attB* site is more sensitive than *attP* to a similar panel of substitutions (Figure 6C). The positions predicted to constitute the ZD motif (T19–C11) show similar disruptions to binding and integration for positions T18, C16 and G15 as observed for the corresponding T23, A21 and G20 of *attP*, but stronger effects are observed for T17 (corresponding to *attP*–T22). The last position in the putative ZD motif of *attB* is also sensitive to substitution. C11G is disruptive to both DNA-binding and integration for *attB*, but T16G is tolerated in *attP*. This result, as well as the disruptive T12G substitution, may be a consequence of overlapping functions required of this region of the *attB* site. Indeed, *attB* base pairs 11 and 12 are involved in binding both the RD and ZD in our model, and their higher sensitivity to substitution may reflect the simultaneous disruption of two, rather than one, protein domain–DNA interfaces. It is also possible that the RD–ZD linker is engaged in the minor groove in this region of *attB*, further increasing the constraints on sequence.

Calos and colleagues (9) have identified 11 pseudo-*attB* sites in the human genome where A118 integrase will integrate *attP*-containing plasmids. An alignment of these sites is shown in Supplementary Figure S6A. Three regions of *attB* conservation are evident: (i) thymidines are strongly favored at positions 17–19, with position 18 nearly invariant; (ii) G15 is strongly conserved and (iii) C11 is strongly conserved. We also note that *attB* positions 13 and 14 are tolerant to substitution for DNA-binding and for integration (Figure 6C), differ between the two LI *attB* half-sites (Figure 6A) and show little sequence preference in the pseudo-*attB* site alignment. The corresponding ZD motif positions in the *attP* site (18 and 19) are also tolerant to substitution, consistent with a lack of contacts to DNA bases in this region by the ZD.

Two important questions to address for our *attB* binding model are (i) can the *attB* sites of other LSR systems be interpreted as having ZD motifs shifted relative to their positions in the *attP* sites and (ii) is a common core complex involving the catalytic and RDs reasonable based on *att* site sequences. For the first question, a summary of putative ZD motifs is shown for several LSRs in Supplementary Figure S6B. In all but one case, a binding motif of 7–8 bp can be readily identified in both the *attP* and *attB* sites. The outlier is the TP901 integrase, where the putative ZD motifs have only weak similarity. This integrase is also unusual in that the RD–ZD linker is slightly longer than is predicted for other LSRs (Figure 3C). Additional residues present in the linker may allow ZDs from LSRs such as TP901 integrase to recognize motifs that are displaced somewhat from that observed for LI integrase, leading to potentially larger contacted *attP* sites. The second question is supported by similarity of *attP* and *attB* sequences in the context of the LI integrase RD-binding region (positions 1–12). This is most evident for the φC31 and Bxb1 integrase attachment sites, whereas the similarity is lower for the A118 sites (Supplementary Figure S6B).

Support for the *attB* binding model shown in Figure 6 also comes from a recent study in the Bxb1 system, where the authors found that simultaneous modifications at positions 14 and 20 of the *attP* half-sites resulted in formation of a functional *attB* site (31). Examination of those substitutions in the context of the site alignments shown in Supplementary Figure S6B suggests that modification at position 14 would disrupt the RD–ZD linker interaction and the change at position 20 would disrupt the ZD–DNA interaction. At the same time, the substitution at position 14 creates a new ZD motif in *attP* that is located at the position predicted for an *attB* site. Thus, *attP* sites may be identified by RD–ZD linker binding segments and flanking ZD motifs and *attB* sites have altered linker-binding sequences and shifted ZD motifs.

To more closely examine the question of structural feasibility, we constructed a model of the LI int–*attB* complex by shifting the ZDs in the *attP* complex 5 bp toward the center of the site (Figure 6D). Importantly, there are no steric clashes imposed by repositioning the ZDs and the interdomain linker conformation can be easily modified to connect the ZD and RD. We did not attempt to model a linker–DNA interaction, but note that the linker could lie in the minor groove as observed in the int–*attP* complex, but running in the opposite direction. The ZD and RD occupy a continuous surface of the *attB* site major groove, extending from positions 9–19. As expected from being translated by 1/2 of a helical turn along the DNA, the ZDs and attached CC motifs in the int–*attB* complex model are oriented in opposite directions relative to those in the int–*attP* complex. Like the *attP* complex, however, the ZDs and CC motifs in the two *attB* half-sites are located on opposite faces of the DNA duplex.

The ZD domains in the int–*attB* complex model are barely close enough that the attached CC motifs could interact (109 Å), perhaps explaining the cooperative binding observed for the isolated φC31 integrase CTD on *attB* sites (38). The central location of the NTD dimer would appear to present a barrier to such interactions when full-length integrase binds to *attB*, but a reduction in the central DNA bend in the site would reduce the ZD–ZD distance somewhat and may facilitate interactions across the bottom of the dimer surface. It is clear from Figure 6D that the CC motifs could easily interact with the catalytic domains when integrase is bound to an *attB* half-site.

### Control of directionality and site-selectivity by LSRs

**Figure 7.**
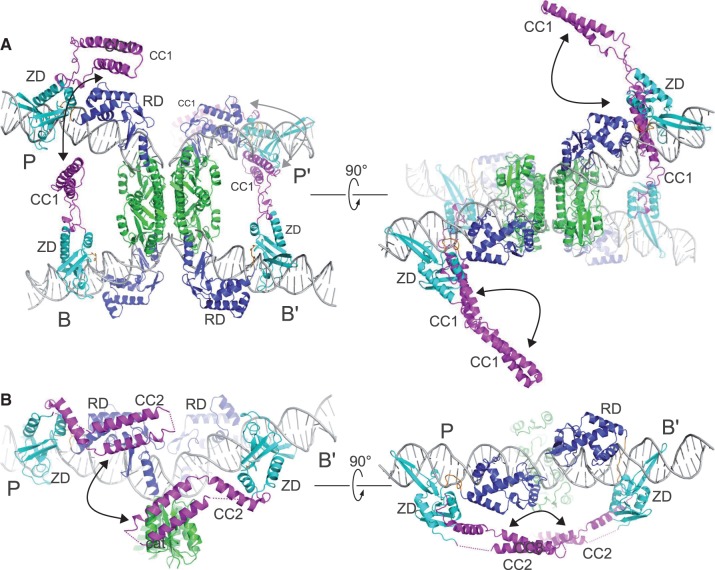
Structural basis for serine integrase directionality. (**A**) Model of an *attP* × *attB* synaptic complex based on the tetrameric γδ-resolvase–DNA synaptic complex (23). The ZDs of juxtaposed half-sites (P and B, P′ and B′) are positioned and oriented so that their attached CC motifs can interact to stabilize the synaptic complex. (**B**) Model of the LI int–*attL* complex, generated from the P and B′ half-sites of int–*attP* (Figure 5A) and int–*attB* (Figure 6D), respectively. The second CC motif conformation (CC2) is shown here, illustrating that the unique ZD arrangements on *attL* and *attR* can facilitate an intramolecular CC–CC interaction.

The CC motif does not have an obvious functional role related to DNA binding and can be removed with no observable loss of *attP* or *attB* binding activity. The CC motif is, however, required for efficient *attP* × *attB* integration (Figure 5D). Indeed, mutations mapping to αK have been demonstrated to dramatically reduce the activity of φC31 integrase, indicating that this region may be involved in important protein–protein interactions (Figure 3C) (39). To investigate whether CC-mediated interactions between *attP*-bound and *attB*-bound integrases are feasible from a structural and architectural standpoint, we constructed a model of a LI int–*attP/* int–*attB* synaptic complex. There are currently no high-resolution structural models available that indicate directly how the associating *attP* and *attB* sites are likely to be spatially arranged at the start of the reaction. However, a tetrameric γδ-resolvase/DNA complex structure representing the next step in the reaction where the DNA has been cleaved provides a good approximation (23). We therefore replaced the DNA half-sites in the resolvase model with LI integrase CTD-bound *attP* and *attB* half-sites to generate the synaptic complex shown in Figure 7A. An alternative model based on the DNA geometry determined in a small angle scattering study of Tn3 resolvase (40) led to similar conclusions as outlined below (see Supplementary Text).

Remarkably, the *attP* and *attB* half-sites that would be juxtaposed during synapsis have their ZDs oriented with an average ZD–ZD distance of 77 Å. In this configuration, the CC motifs from the P and B half-sites could interact and the CC motifs from the P′ and B′ half-sites could interact based on the positions and orientations of their ZDs and a conservative estimate of 54 Å for the length of the CC motif. Interactions between CC motifs in the int–*attB* half-sites and either the NTDs, RDs or ZDs in the int–*attP* half-sites are also possible. Note that the P-B and P′-B′ half-site pairs are expected to rotate with respect to one another during strand exchange (15) and therefore any CC-mediated interactions within these pairs could remain intact throughout this process. An alternative arrangement with CC-mediated interactions between P-B′ and P′-B half-sites is not feasible because the ZDs involved are too far apart (142 Å) and are improperly oriented. This makes mechanistic sense because diagonal interactions across the tetramer interface would be expected to inhibit strand exchange by preventing subunit rotation.

In the serine integrase systems that have been studied, *attP* × *attP* and *attB* × *attB* recombination are inefficient (2,33). To ask how the corresponding complexes might differ from *attP* × *attB*, we constructed models of two synaptic int–*attP* complexes and of two synaptic int–*attB* complexes (Supplementary Figure S7). In the *attP* × *attP* model, the distances between ZDs are greatly increased (154 Å) relative to those predicted for the *attP* × *attB* complex and consequently, interactions between CC motifs do not appear likely. The situation for the *attB* × *attB* complex formation is different; the ZD–ZD distances (∼35 Å) are even closer than those predicted for the *attP* × *attB* complex. Given the close proximity of the ZDs in juxtaposed half-sites, interactions between CC motifs would have to be different from those formed in the *attP* × *attB* complex, if they can form at all. It also seems likely that steric interference could play a role in inhibiting *attB* × *attB* synapsis. The segments bridging the ZD and the CC motif are poorly ordered in our structural models, but given their approximate locations they could act as a barrier to *attB* × *attB* association. Thus, for self-association of *attP* sites and *attB* sites, the arrangements of ZDs in the participating half-sites differ significantly from those in the *attP* × *attB* complex. We suggest that these alternative arrangements do not permit interactions between CC motifs that coincide with formation of an activated catalytic domain tetramer that can undergo strand exchange.

The *attL* × *attR* reaction is somehow blocked by the integrase CC motifs (Figure 5C), despite the argument that productive interactions between P and B arms and between P′ and B′ arms of the *attP* × *attB* complex are likely to exist through formation of *attL* and *attR*. To examine this apparent paradox more closely, we constructed an int–*attL* model by combining the P and B′ half-sites of the int–*attP* and int–*attB* complexes, respectively. As shown in Figure 7B, the ZDs in the int–*attL* complex are positioned and oriented such that their CC motifs could easily interact with one another. The CC motif trajectories required for these intramolecular interactions are different from those needed to form intermolecular interactions between juxtaposed half-sites in the *attP* × *attB* model. Interestingly, one of the four CC conformations observed experimentally appears particularly suitable for promoting a CC–CC interaction within the *attL* complex (shown in Figure 7B). The *attR* complex has an identical arrangement of ZDs, but on the opposite face of the DNA duplex (Supplementary Figure S7C).

**Figure 8.**
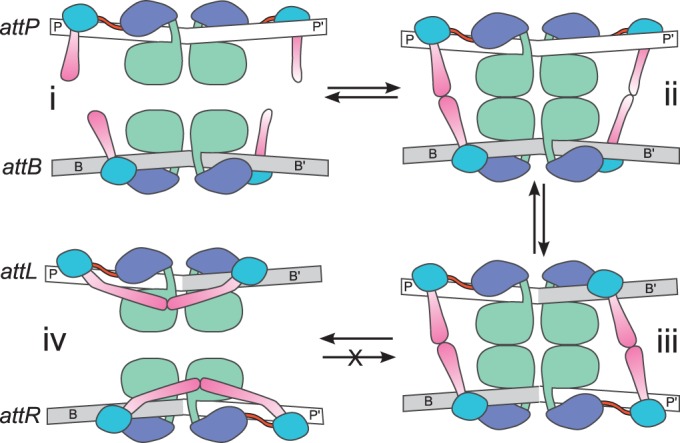
Schematic model of serine integrase regulation of directionality. Integrase-bound *attP* and *attB* sites (i) associate to form a synaptic complex (ii) that is stabilized by CC-mediated interactions unique to the *attP* × *attB* site pairing. Cleavage, subunit rotation and strand exchange result in a product synaptic complex of *attL* and *attR* sites (iii) that are able to form autoinhibitory interactions between CC motifs that compete with those required for the reverse excision reaction (iv).

The int–*attL* complex shown in Figure 7B suggests a mechanism for inhibition of *attL* × *attR* recombination where CC-mediated interactions stabilize synapsis-incompetent configurations of the int–*attL* and int–*attR* complexes. These intrasite interactions could be established on dissociation of the *attL* × *attR* complex formed during the *attP* × *attB* integration reaction and could play an active role in disassembly of the product complex. This model explains why serine integrases do not efficiently recombine *attL* or *attR* sites with themselves or with other sites in the absence of a RDF protein. A schematic mechanism illustrating how CC-mediated interactions between half-sites could regulate site selectivity and directionality of recombination is shown in Figure 8.

## DISCUSSION

Differences in the way serine integrases bind to *attP* vs. *attB* lie at the heart of the enzyme’s ability to selectively catalyze recombination between those sites but not between any other pairing of sites derived from the same half-site components. The work described here indicates that these differences are not subtle. We propose that the *attP*- and *attB*-bound integrases share a common core architecture involving the catalytic domain dimer, αE and RD but differ dramatically in the quaternary arrangement of their ZDs. The ZD organization and consequently the spacial arrangement of CC motifs on the different attachment sites forms the structural basis for regulation of directionality.

In the model shown in Figure 8, the CC motif acts as both a positive and negative regulator of synapsis. Positive regulation occurs when CC-mediated interactions between integrases bound to attachment sites are able to augment and stabilize the tetrameric interface between two catalytic domain dimers that is required to activate cleavage and strand exchange. Negative regulation occurs through autoinhibition of int–*attL* and int–*attR* complexes as a result of the unique half-site pairing and ZD proximity that these sites possess. The *attB* sites are also negatively regulated for self-association. When the CC motifs are removed, the attachment sites become functionally similar and can be recombined promiscuously under conditions where synapsis is otherwise facilitated. Thus, the LSR enzymes have the domain architecture and catalytic machinery of the resolvase/invertases at their core (NTD and RD), and it is the presence of the ZD and CC motif that provide LSR-specific functionality.

In the φC31 and Bxb1 integrase systems, it has been demonstrated that efficient synapsis and recombination are only observed between int–*attP* and int–*attB* sites in the absence of a phage-specific RDF (16,17). In principle, int–*attL* and int–*attR* complexes should also be able to form a stable synaptic complex because they can form the same productive P–B and P′–B′ interactions that led to their formation. We suggest that these int-bound sites do not associate because they form intramolecular interactions between CC motifs that compete with the intermolecular contacts required for stable synapsis. These autoinhibitory interactions would be expected to block subunit rotation even if transient synaptic complexes were to form via interactions between catalytic domain dimers. It is also possible that intramolecular interactions involving the CC motifs inhibit synapsis through additional conformational changes. These ideas may explain why WT LI integrase is inactive for *attL* × *attR* excision in the same assay where the ΔCC enzyme is moderately efficient. The *attL*/*attR* model also provides an explanation for cooperative binding observed by isolated φC31 integrase CTDs on *attL* and *attR* sites (38). Indeed, the idea that the CC motif region is somehow sequestered when φC31 integrase is bound to *attL* and *attR* was inferred from biochemical data (39) and can now be further explored with aid of a structural framework.

Hyperactive mutants in φC31 integrase have been identified that gain the ability to carry out *attL* × *attR* recombination in the absence of the φC31 RDF (39). These substitutions map to the CC motif where several acidic residues are predicted to lie on the same face of an extended αK (Figure 3C and Supplementary Figure S3). Because these residues are located near the base of the helical dimer, they may be associated with positioning of the CC motif rather than with a surface used in interactions between CC motifs. We suggest that these hyperactive mutants affect the range of trajectories accessible to the CC, destabilizing the autoinhibitory interactions that are unique to the *attL* and *attR* sites. Because our models predict that the CC conformations responsible for stabilizing *attP* × *attB* synapsis are different, this could explain why the hyperactive mutants do not disrupt the integration reaction.

The structural models described here shed light on possible roles of the RDFs responsible for stimulating *attL* × *attR* recombination. Two excision-stimulating proteins have been studied in some detail: gp47 for mycobacteriophage Bxb1 (41) and gp3 for bacteriophage φC31 (42). These proteins bear no resemblance to one another at the sequence level, but both interact with their respective integrases to stimulate excision. A simple mechanism for RDF function could involve blocking the intramolecular CC motif interactions that prevent integrase-bound *attL* and *attR* from associating. This could be accomplished by RDF binding to the ZD and/or to the CC motif. However, the RDF also inhibits *attP* × *attB* recombination in the Bxb1, φC31 and φRV1 systems (36,41,42). Understanding how the RDF stimulates excision while inhibiting integration will require further information on the nature of the RDF–integrase interactions, but in principle an inhibitory RDF–integrase interaction forming on *attP* or *attB* could take advantage of the unique ZD arrangements on those sites.

An additional aspect of the synaptic complex architecture that emerges from our structural model concerns the symmetric nature of the *attP* × *attB* reaction and the asymmetric nature of the *attL attR* reaction. The *attP* × *attB* complex model has a 2-fold–symmetric arrangement of ZDs with respect to an axis passing through the central dinucleotides of the attachment sites (Figure 7A). This is consistent with findings that the sites can be synapsed, cleaved and undergo subunit rotation equally well in either a parallel or antiparallel orientation with respect to the central crossover dinucleotide, but only the parallel alignment will result in efficient strand exchange (43,44). The *attL* × *attR* complex, on the other hand, has distinct domain arrangements for parallel vs. antiparallel site alignments. The productive parallel alignment has all four ZDs on the same face of the synaptic complex, whereas the antiparallel alignment results in P–P′ and B–B′ half-sites juxtaposed (Supplementary Figure S7C). This aspect of the model explains the parallel *attL* × *attR* and antiparallel *attL* × *attL*/*attR* × *attR* site alignment preferences observed for Bxb1, φC31 and φRV1 excision in the presence of their RDFs (42,45). The same alignment preference was observed for excision by hyperactive φC31 mutants in the absence of the RDF, indicating that this asymmetry is a property of the int–*attL* and int–*attR* complexes and is not imposed by the RDF (39). Overall, the structural models described here provide strong support for the idea that two sets of interactions between P-derived and B-derived half-sites are required for formation of a stable synaptic complex (39,45). This can be accomplished by antiparallel or parallel *attP* × *attB* alignments, parallel *attL* × *attR* alignment or antiparallel *attL* × *attL* and *attR* × *attR* alignments.

Because several of the serine integrases function well in mammalian cells and in some cases show promise for applications such as gene therapy, there is substantial interest in understanding DNA-binding specificity on a level sufficient for altering specificity through protein engineering (46,47). The work described here is an important step in that direction for the A118 integrase and identifies the residues and DNA-binding motifs that should be targeted when *in vitro* evolution strategies are employed for all serine integrases. Higher resolution structures of the int–*attP* interface and complementary structures of int–*attB* complexes remain important goals and should further facilitate efforts to understand and modify serine integrase site recognition. Because some LSRs are involved in the movement of bacterial resistance markers via transposition and mobile cassette elements, these models may also be useful frameworks for targeting antibacterial therapeutics.

## ACCESSION NUMBERS

PDB code 4KIS.

## SUPPLEMENTARY DATA

Supplementary Data are available at NAR Online, including [48–57].

Supplementary Data
