# Supplementary material for: Attachment site recognition and regulation of directionality by the serine integrases
Source: Nucleic Acids Res. 2013 Jul 2;41(17):8341–56. doi: 10.1093/nar/gkt580 (PMC3783163; doi:10.1093/nar/gkt580)
Supplement: Supplementary Data [file supp_41_17_8341__index.html]

Attachment site recognition and regulation of directionality by the serine integrases — Attachment site recognition and regulation of directionality by the serine integrases — Supplementary Data 

# Attachment site recognition and regulation of directionality by the serine integrases

## 

files

**Files in this Data Supplement:**

- Supplementary Data - pdf file
