# Supplementary material for: Attachment site recognition and regulation of directionality by the serine integrases
Source: Nucleic Acids Res. 2013 Jul 2;41(17):8341–56. doi: 10.1093/nar/gkt580 (PMC3783163; doi:10.1093/nar/gkt580)
Supplement: Supplementary Data [file supp_gkt580_nar-01493-h-2013-File010.pdf]

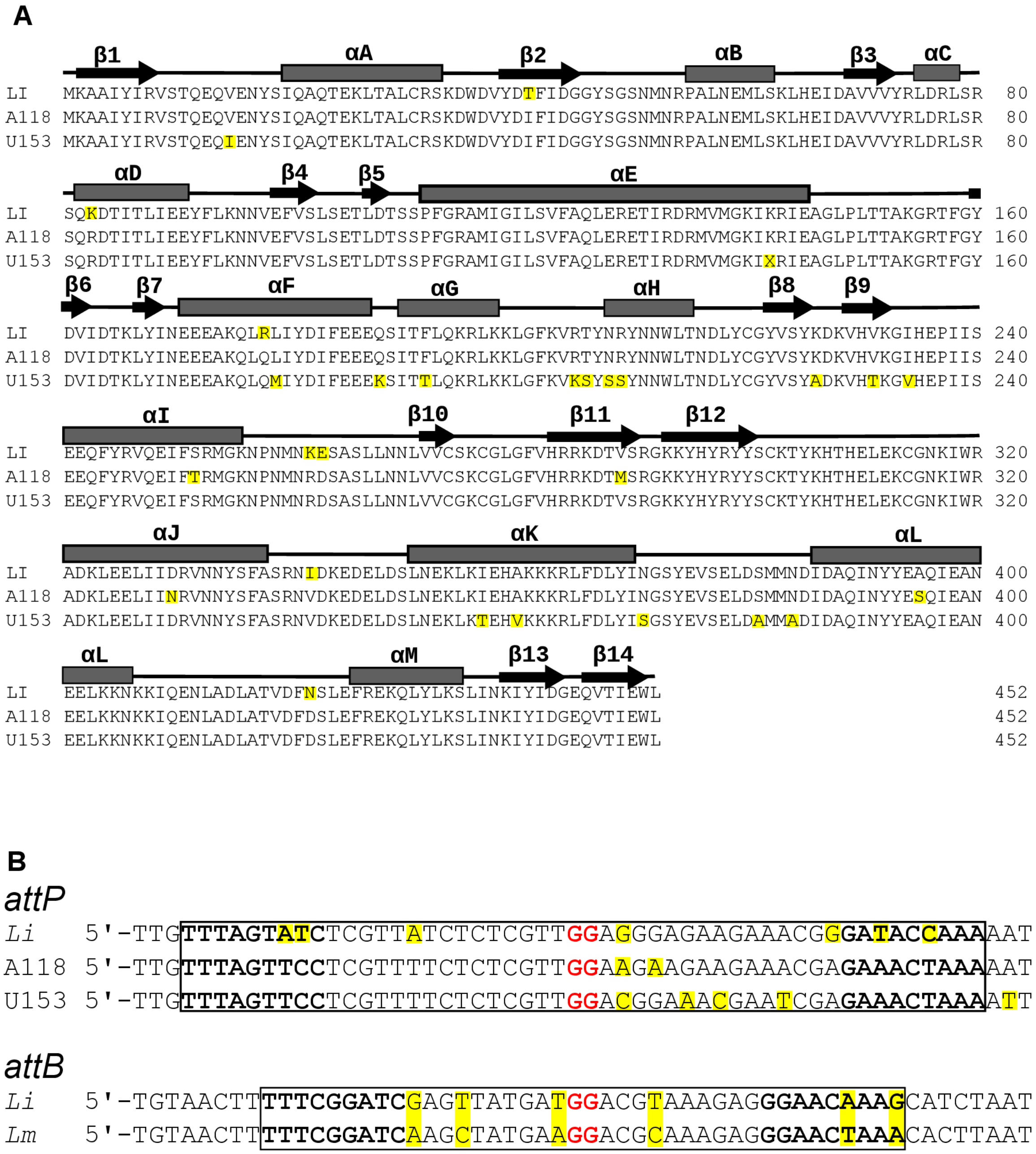

Supplemental Figure 1. Alignments of *Listeria* phage integrases and attachment sites. A) Sequence alignment of the integrases from lysogenic *Listeria innocua* strain CLIP 11262 (NCBI accession # NP_471756), bacteriophage A118 (NP_463492), and bacteriophage U153 integrase (CAD10283). Secondary structure assigments from the TP901 integrase (31) are shown for residues 1-132 and secondary structure assignments from the LI integrase/attP structure described here are shown for the remainder. Residues that differ among the three sequences are highlighted in yellow. B) Alignment of *attP* sites for the three integrases compared in (A) and two *Listeria attB* sites. The integrase-DNA contact region is boxed and the zinc domain binding motifs are in boldface (predicted for *attB*). The crossover site is colored red and differences between the sites are highlighted in yellow. The *attB* alignment shown is for *Listeria innocua* and the *attB* site from *Listeria monocytogenes* strain SLCC5850 (FR733647). The *attP* and *attB* sequences for *Listeria innocua* were deduced from the *attL* and *attR* sequences.

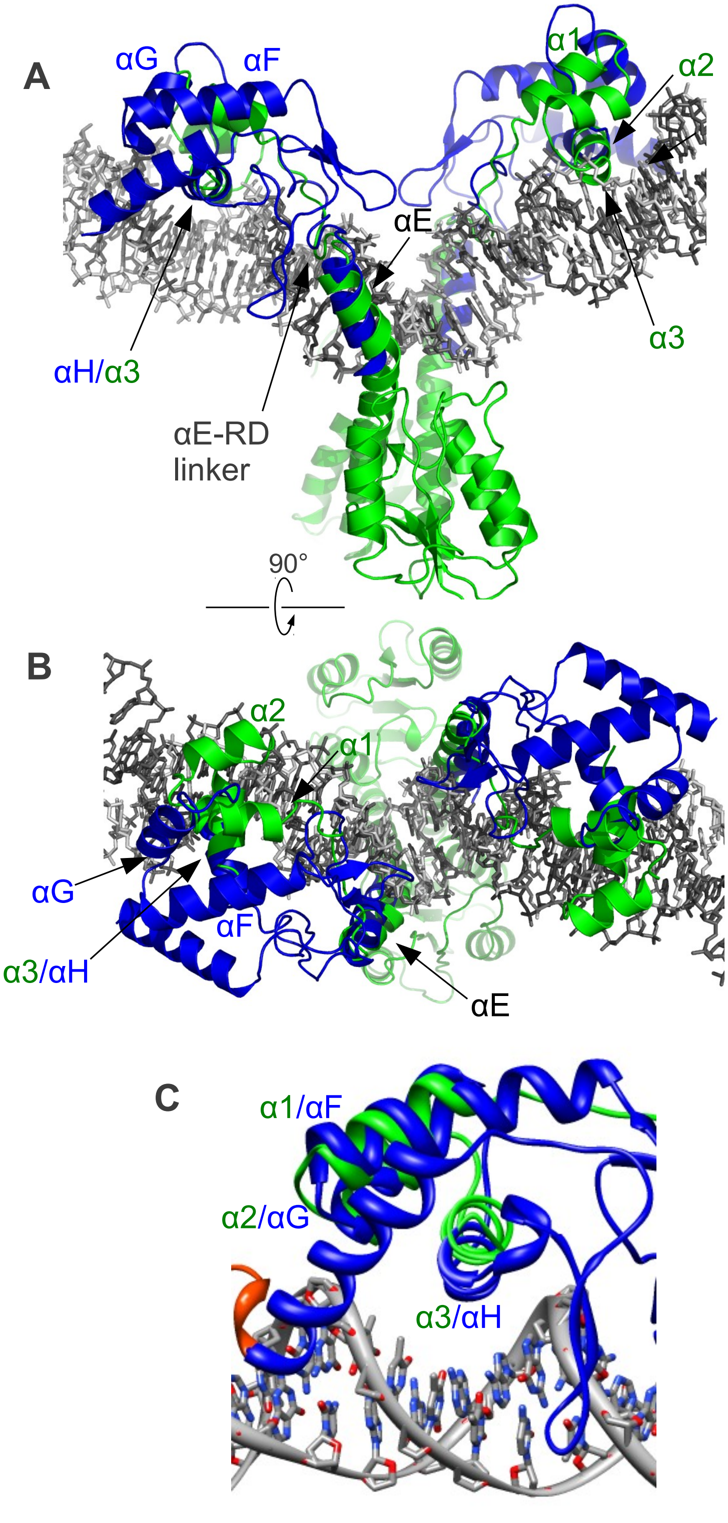

Supplemental Figure 2. Similarity of LI integrase RD with γδ-resolvase. A) Superposition of the LI integrase/*attP*-HS complex onto the half-sites of γδ-resolvase/*res* complex (pdb 1GDT). B) Orthogonal view of that shown in (A). The resolvase/*res* complex is in green and gray and the LI integrase/attP complex is in blue and black. The superposition was performed based on the innermost five base-pairs of the corresponding half-site DNAs, not including the crossover dinucleotide. The zinc ribbon domains and the extended DNA of the attP site are not shown. The three resolvase helices labeled α1, α2, and α3 make up the helix-turn-helix domain in the resolvase/invertase enzymes. The corresponding helices in the LSR enzymes are αF, αG, and αH, respectively, which form a similar helix-turn-helix core domain. There is some overlap between α3 and αH, but the three-helix bundle is rotated in the integrase-*attP* complex to lie in a different orientation and position in the major groove relative to that observed in the resolvase complex. Note the similar orientations and positions of both αE and the linker following αE. C) Superposition of the resolvase HTH motif (green) onto the αF-αG-αH helical bundle in the LI integrase recombinase domain.

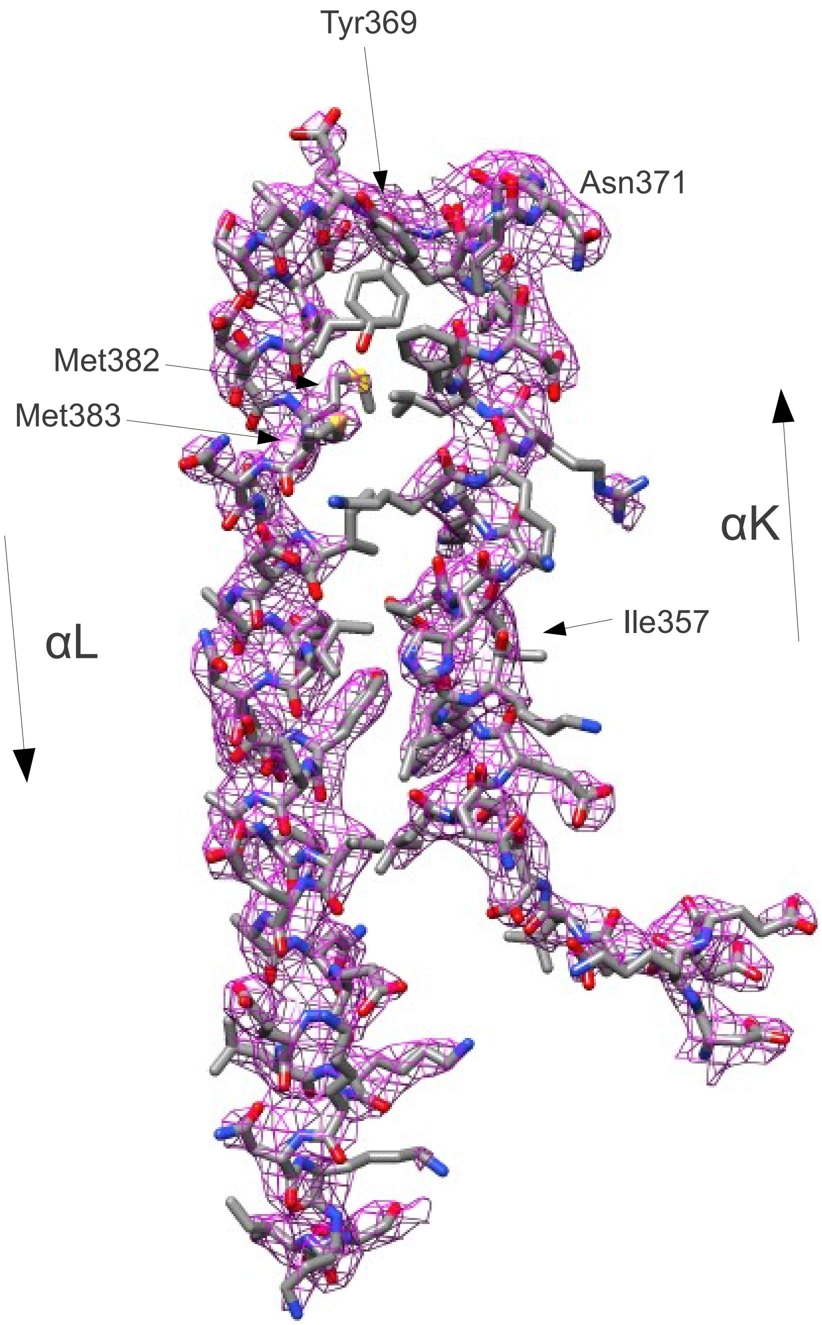

Supplemental Figure 3. Electron density for the coiled-coil motif. A segment of the CC1 helical region is shown. The density is from a weighted $2F_o$-F_c map at 3.2 Å, contoured at 1.5σ. Ile357 aligns to Glu463 in φC31 integrase, one of the sites where hyperactive mutants have been identified. Tyr369 aligns to Tyr475 of φC31 integrase, where the histidine substitution has been shown to strongly decrease recombination efficiency for all site pairs (38).

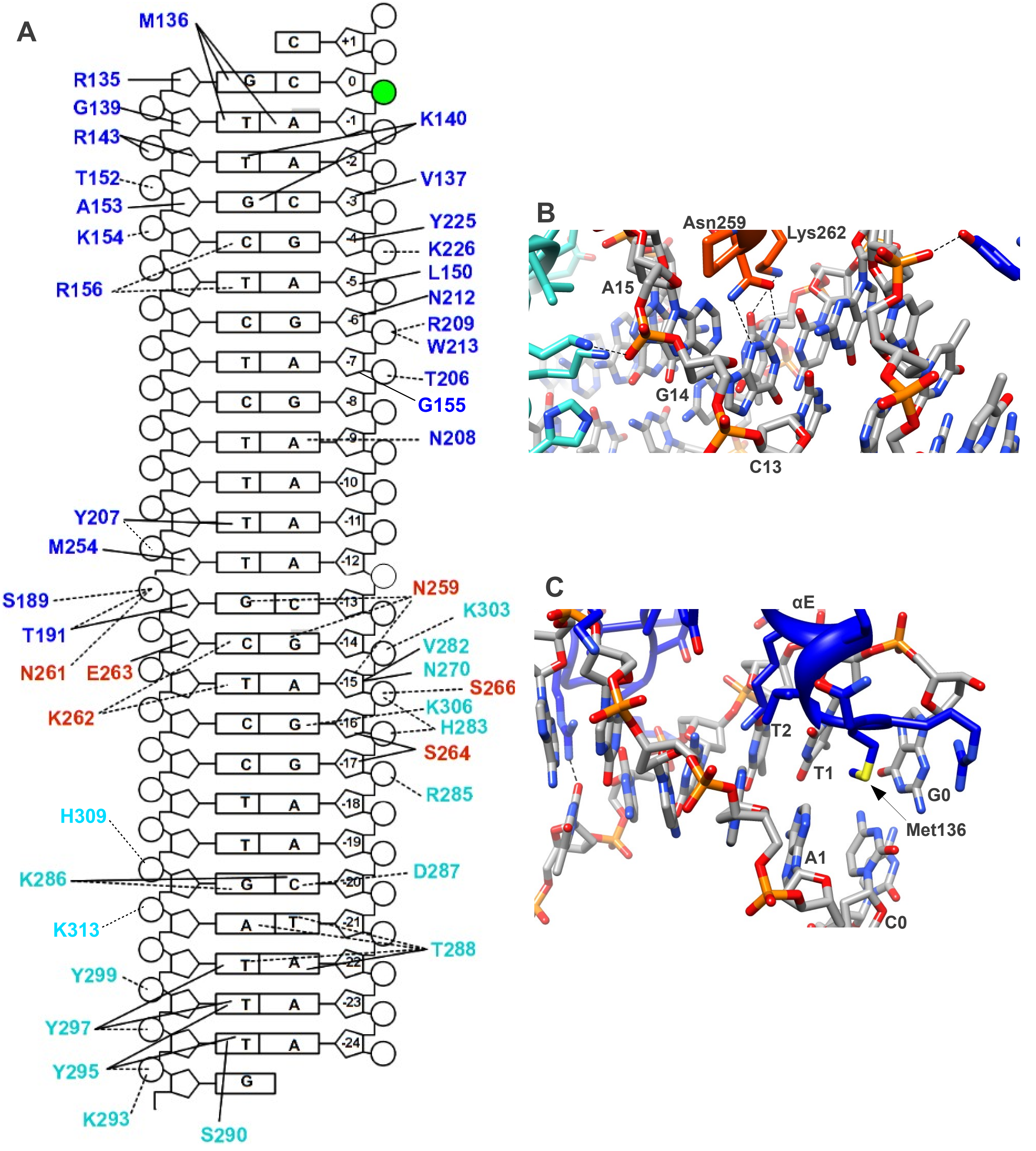

Supplemental Figure 4. Integrase-*attP* interactions. A) Schematic of LI integrase CTD-*attP* half-site contacts. Base-pairs are numbered as in Fig. 4. Residue labels are color-coded based on domain structure, as used in the main text (blue: RD; cyan: ZD; red: RD-ZD linker). Dashed lines indicate hydrogen-bond interactions and solid lines indicate van der Waals contacts. The scissile phosphate is colored green. B) Closeup of the Asn259-minor groove interaction in the RD-ZD linker. C) Closeup of Met136 from αE intercalating between the G0 and T1 bases in the minor groove.

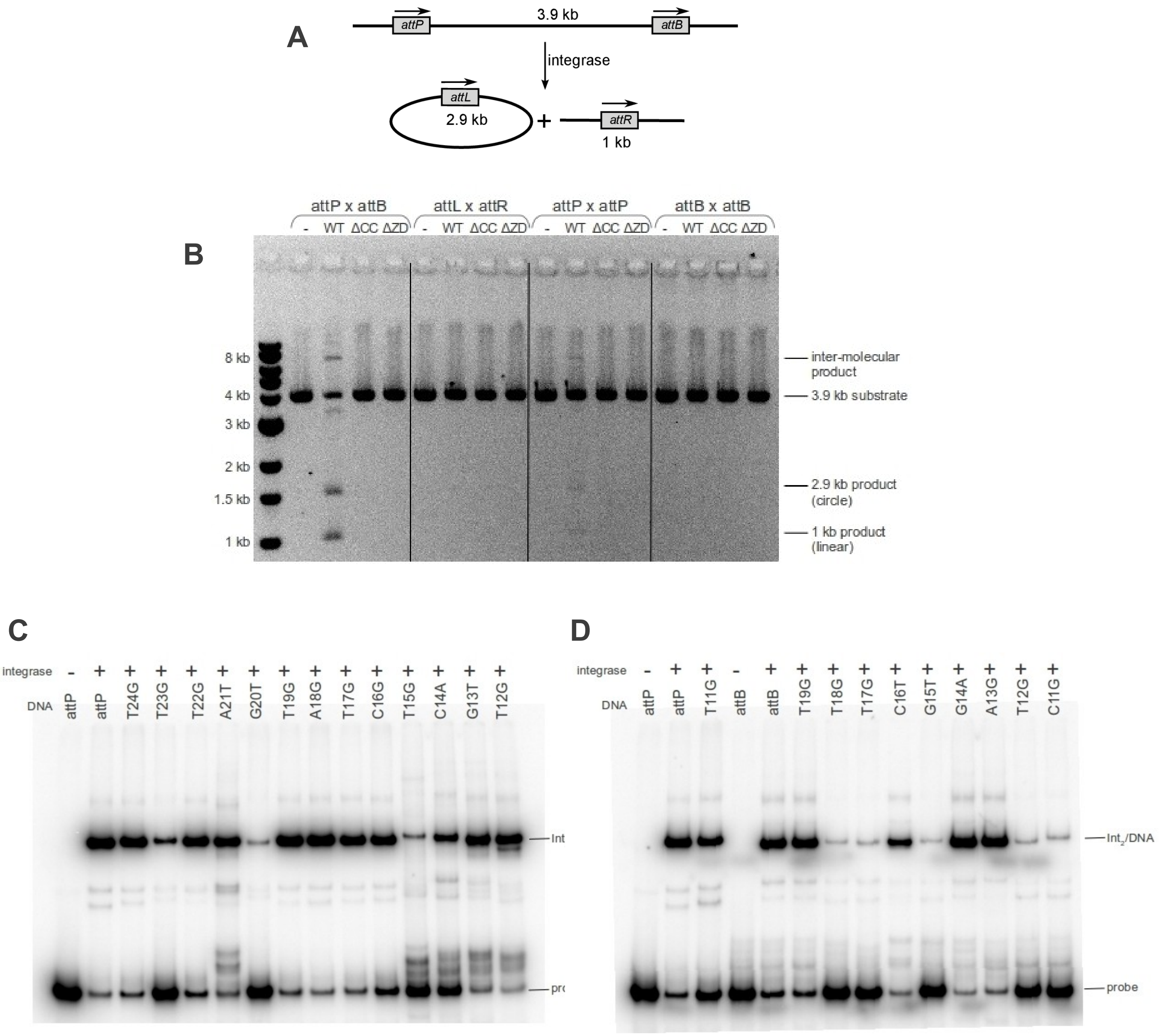

Supplemental Figure 5. *In vitro* recombination and DNA-binding assays. A) Schematic of the in vitro recombination reaction. A linearized plasmid containing phage A118 attachment sites in direct repeat (with respect to the crossover dinucleotide) is converted to 2.9 kb circular and 1 kb linear products by wild-type LI integrase. B) *In vitro* recombination reactions containing 3.9 nM substrate and 100 nM integrase were incubated 120 min at 27°C and then separated on 0.8% agarose containing ethidium bromide. Wild-type integrase converts the attP x attB substrate to products and weakly recombines the attP x attP substrate but no products are observed for attL x attR or attB x attB. No products were observed for the ΔCC or ΔZD integrases in this assay. The 2.9 kb circular product runs as 1.7 kb in the presence of ethidium bromide. This assay was repeated several times with various buffers, temperatures, and reaction times, with similar results. See supplemental methods for final buffer composition. C) and D) DNA-binding of LI *attP* and *attB* mutants by LI integrase using electrophoretic mobility shift assays. DNA probes are 119 bp in length and contain 56-bp attP or attB sites. Modified sites all contain symmetric substitutions in both half sites. DNA-binding reactions contained ~1 nM DNA and 50 nM integrase and were separated on 6% polyacrylamide at 15°C using Tris-glycine buffer, pH 8.3. DNA binding was scored +++ for near wild-type binding, ++ for slightly defective, and + for severely defective, as shown in in Figs. 6B & 6C.

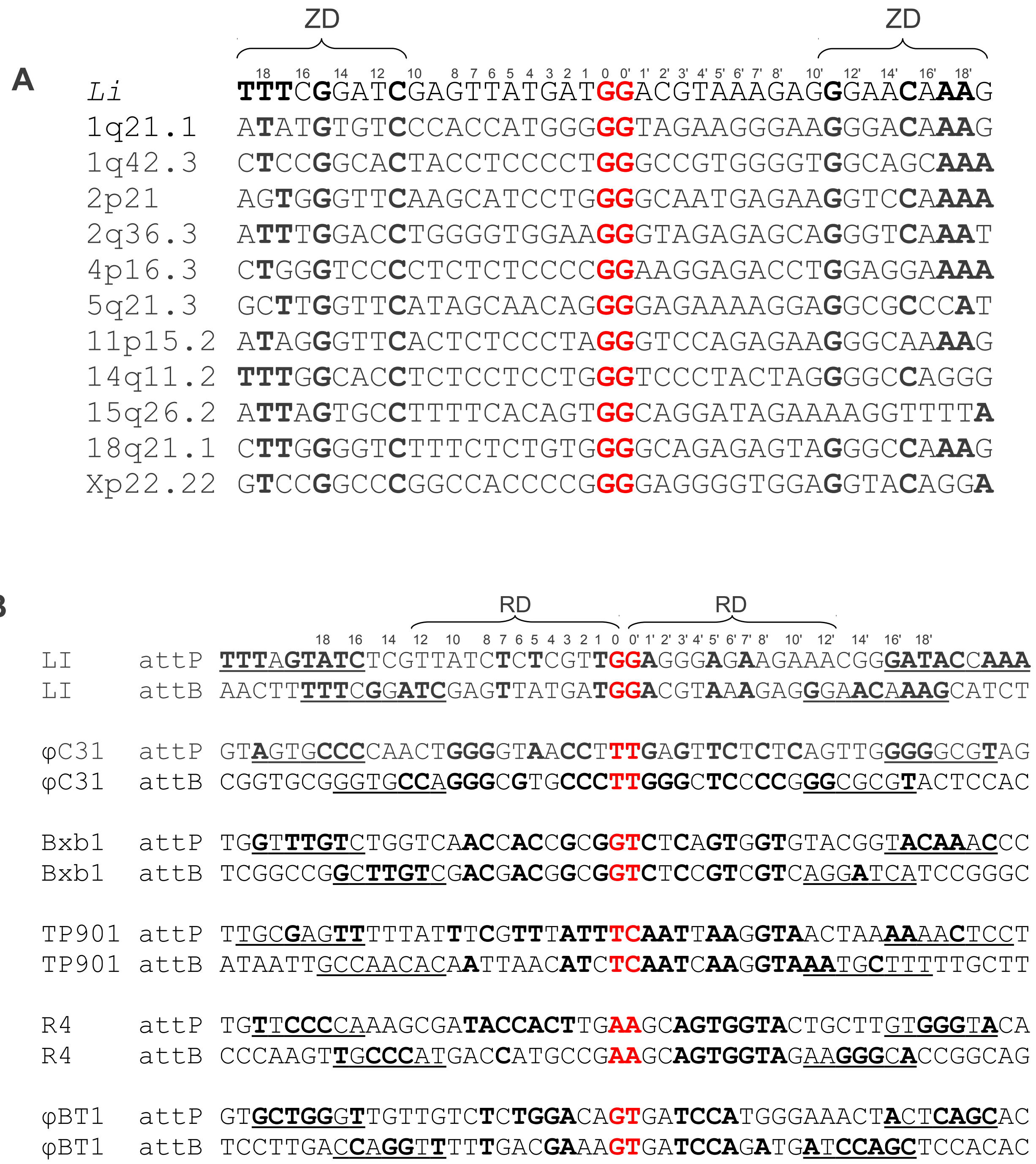

Supplemental Figure 6. Human pseudo-*attB* sites and other LSR attachment sites. A) Alignment of eleven pseudo-*attB* sites from the human genome (9). The sites were identified from integration of A118 *attP*-containing plasmids by bacteriophage A118 integrase. Chromosomal locations are given for each site and the *Listeria innocua attB* site is shown for reference. Numbering follows that used in Figure 6 of the main text. The putative zinc domain binding motifs are indicated. Residues that show high similarity to the putative ZD motif in LI *attB* are in bold. B) Attachment sites for several serine integrases, with predicted zinc domain binding motifs underlined. Residues that are identical in three or four of the four ZD motifs within a given system are in bold. Residues within positions 1-10 of the recombinase domain (RD) binding region that are present in three or four of the four half-sites are also in bold. The central crossover dinucleotides are in red. The systems shown are for bacteriophage A118/LI (this work), φC31 (2), Bxb1 (16), TP901 (57), R4 (56), and φBT1 (36).

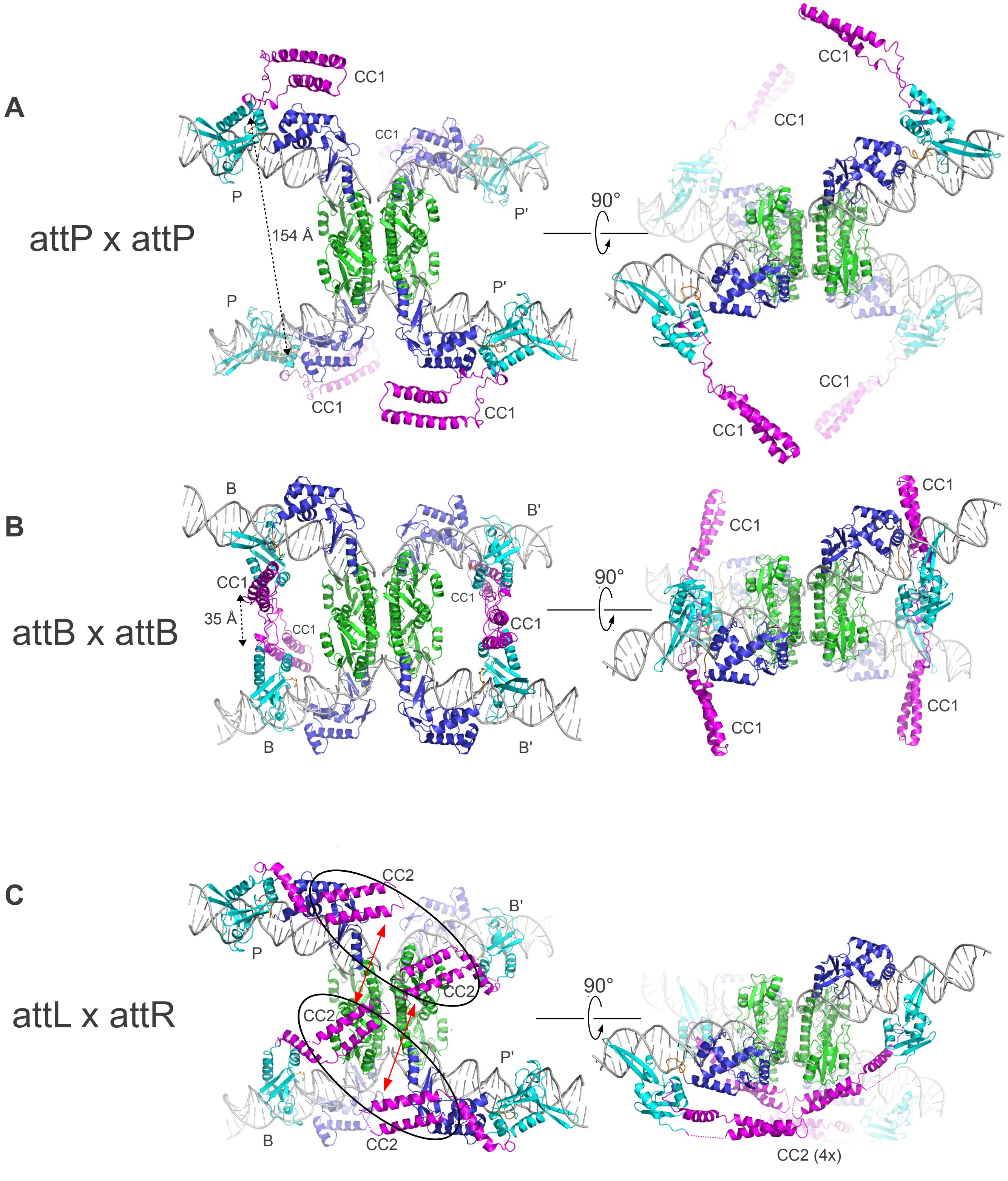

Supplemental Figure 7. Models of weakly formed LI integrase synaptic complexes. A) *attP* x *attP* complex. The zinc domains are located on opposite faces of the synaptic complex for juxtaposed half-sites and are ~154 Å apart (meausured from Ala338). B) *attB* x *attB* complex. The zinc domains are ~35 Å apart, with evidence for steric interference between ZDs. The streric clash worsens for smaller crossing angles between the synapsed sites. C) *attL* x *attR* complex, in the parallel orientation with respect to the crossover dinucleotide. Note that all four CC motifs are on the same face of the synaptic complex. Red arrows indicate CC-CC interactions predicted to form in the active synaptic complex (i.e., the same as would exist during *attP* x *attB* recombination). The CC motifs involved in competing autoinhibitory interactions that are predicted to form on *attL* and *attR* are circled. CC1 and CC2 refer to the CC motifs observed in the first and second independent molecules in the crystal structure, respectively.

Supplemental Methods

Reagents and Strains. Strains CSH100 (F' *ara* Δ(*gpt-lac*)5) and CSH142 (F^- *ara* Δ(*gpt-lac*)5) were obtained from the *E. coli* Genetic Stock Center (CGSC8105 and CGSC8083) at Yale University. Plasmids pFW11 and pTSA29 were gifts from Fred Whipple and Gregory Phillips, respectively.

Generation of LI integrase expression constructs. An expression construct of the WT LI integrase (residues 1-452) was prepared by polymerase chain reaction (PCR) amplification of the coding region from *Listeria innocua* genomic DNA (ATCC BAA-680D) and ligation into pET29b (Novagen). The WT construct served as a template for generating the remaining constructs used in this study by PCR. The S10A catalytic mutant was created by inverse pcr mutagenesis. The ΔCC construct (Δ342-416) was created by four-primer pcr to delete residues 342-416. The CTD (133-452) and ΔZD (1-264) constructs were sub-cloned from the WT expression construct. PCR products were digested with NdeI and XhoI restriction enzymes and ligated into the same sites of pET29b for expression and purification or into the same sites of pACYCBad1 for arabinose-inducible expression in *in vivo* assays. pACYCBad1 has the pACYCDuet backbone (Novagen), where an *E. coli araC*-P*BAD* cassette has been PCR-cloned to replace the two T7-promoters. The CTD construct for crystallizaton was ligated into pETDuet (Novagen) in frame with a C-terminal His_6 tag.

LI integrase expression and purification. The WT, S10A, ΔCC, ΔZD and CTD-His_6 expression plasmids were transformed into BL21(DE3) cells and overexpressed in LB broth supplemented with 100 µM zinc sulfate and IPTG induction at 18ºC for 16 h. For selenomethionine (SeMet) labeling, the CTD construct was overexpressed in BL21(DE3) cells at 18°C in minimal media containing 125 µg/ml L+-SeMet and 10 ug/ml unlabeled methionine as the sole methionine sources, and 0.1 µM Vitamin B12, as described (19), except that α-lactose was omitted from the minimal media to prevent auto-induction.

CTD-His_6 was purified using Ni-NTA (QIAGEN) beads following the vendor protocol, followed by dialysis vs. 20 mM MES pH 6.5, 0.4 M NaCl, 2.5% glycerol and 5 mM 2-mercaptoethanol, and MonoS cation exchange chromatography (GE Healthcare) using a sodium chloride gradient at pH 6.5. Lysates for the WT, S10A, ΔCC, and ΔZD constructs were loaded onto a 20 ml SP-Sepharose (GE) column and eluted using a sodium chloride gradient at pH 8.0, followed directly by injection onto a 5 ml type II hydroxyapatite column (BioRad) and elution using a phosphate gradient at pH 8.0. The final purification step for all proteins was gel filtration using a Superdex 200 column (GE) in 20 mM Tris 7.4-8.0, 500 mM NaCl, and 5 mM 2-mercaptoethanol. Proteins were concentrated at 4ºC using Amicon ultra-centrifugal filter units (Millipore), dialyzed into a storage buffer containing 10 mM TrisCl pH 7.0-8.0, 500 mM NaCl, 1 mM TCEP, and 30% glycerol, and aliquots were flash-frozen in liquid nitrogen for storage at -80ºC. Proteins were assessed as > 95% pure by Coomassie-stained SDS-PAGE. CTD-His_6 is monomeric based on size-exclusion chromatography coupled to multi-angle light scattering analysis.

Oligonucleotide synthesis and purification. The DNA construct used for crystallization is derived from the phage A118 *attP* P-arm as shown below:

```
5'-GTTTAGTTCCTCGTTTTCTCTCGTTG-3'
```

3'-AAATCAAGGAGCAAAAGAGAGCAACC-5'

Head-to-tail stacking in the crystal with formation of G-C base pairs using the 5'-G and 5'-C overhangs generates the second G in the central dinucleotide crossover of the *attP* site. The oligonucleotides used in crystallization were synthesized on a MerMade 4 oligonucleotide synthesizer (Bioautomation) and purified using Glen-Pak reverse-phase cartridges (Glen Research), followed by concentration and buffer exchange into 10 mM TrisCl pH 8.0, 100 mM NaCl using Centricon-3 devices (Millipore). Oligonucleotides were annealed in 10 mM Tris pH 8.0, 100 mM NaCl by slow cooling of a water bath from 100 to 20°C.

Crystallization of the CTD-*attP* half-site complex. CTD and *attP* half-site substrate were mixed in a 1:1.5 stoichiometry in 10 mM TrisCl pH 8.0, 100 mM NaCl, 1 mM TCEP and incubated at 20°C for a minimum of one hour prior to crystallization. Crystals were grown by hanging drop vapor diffusion from initial drops containing 30 µM CTD, 45 µM DNA, 25 mM sodium HEPES pH 7.0, 50 mM NaCl, 13 mM $CaCl_2$, 9% 2-methyl-2,4-pentanediol (MPD), 3% glycerol, and 0.5 mM TCEP that were equilibrated against reservoirs containing 50 mM sodium HEPES pH 7.0, 26 mM $CaCl_2$, 17% MPD, and 6% glycerol. Cubic crystals (I23; a=290.8 Å) grew after 3 days at 21°C and were flash cooled directly in liquid nitrogen. The crystals contained zinc intrinsically. Crystals grown using the LI integrase CTD and the A118 *attP* P-arm half-site were better in terms of growth and diffraction compared to other combinations involving the LI and A118 integrase CTDs and the LI and A118 *attP* P and P' half-site sequences. These differences were not rigorously tested by repeated purifications, crystallizations, and diffraction measurements.

Structure Solution and Refinement. Diffraction data for the native and SeMet crystals were measured at the Advanced Photon Source NECAT 24-ID-C and 24-ID-C beamlines, respectively,and processed with the HKL suite (48). Two native and five SeMet datasets were merged to improve completeness and to boost the anomalous signal (49). The programs ShelxCDE and SOLVE as implemented in PhenixAutosol (50) were used to locate Se and Zinc sites. The SeMet sites were used to phase the data for the CTD-DNA complex at 5.3A, where the anomalous signal was strong, and density for the DNA lattice and recombinase domain were readily visible in the solvent-flattened electron density maps. The CCP4 program DM (51) was used to extend the phases to 3.5 Å using four-fold averaging of the RD, ZD, and DNA, and flattening of the 75% solvent content. The resulting electron density maps were exceptionally clear and allowed fitting of the RD, ZD, and DNA. The CC motifs were fit into electron density following refinement and contain several poorly ordered segments in each of the four independent complexes. The first CC motif (CC1) could be confidently assigned sequence with the aid of methionine positions verified by anomalous difference maps. Iterative model building/adjustment in COOT (52) and rounds of refinement with CNS (53) with NCS restraints yielded a final refined model at 3.2 Å with R_{work} = 0.234 and R_{free} = 0.256. The final model includes the entire *attP* half-site 26-mer and the following integrase residues: 134-460 for chain A, 134-338, 347-369, 377-455 for chain B; 134-369, 381-411, 416-452 for chain C; and 134-340, 344-355, 386-410, and 421-460 for chain D. 82, 15, 2, and 0.7 percent of residues lie in the preferred, additionally allowed, generously allowed, and disallowed regions of the Ramachandran plot, respectively, not including the poorly ordered regions of CC motifs. In addition to the zinc ions present in each of the four ZDs, two additional zinc sites were identified in anomalous difference maps and were included in the refinement. These zinc ions are coordinated by histidine residues in the C-terminal His_6 tags

of two of the four molecules in the asymmetric unit.

Generation of *attP* and *attB* mutants. *attP* and *attB* ultramers (56-mer att sites flanked by 21-bp and 18bp segments; IDT) were amplified and subcloned into the BamHI and HindIII sites of pFBR6kamp, an R6kγ plasmid containing an ampicillin marker (G.V., unpublished). This plasmid can only replicate in strains containing the *pir* gene. Mutants were created by inverse PCR mutagenesis using a long, phosphorylated forward primer (IDT) containing both sequence changes and a short, unphosphorylated reverse primer. Plasmids were transformed into strain PIR1 (Invitrogen) and sequenced.

DNA binding assays. DNA probes for electrophoretic mobility shift assays (EMSAs) were 119-mer duplexes containing 56-bp *attP* or *attB* sequences. ^{32}P-labeled probes were generated by PCR amplification of cloned sites followed by purification on Centri-Spin-20 columns (Princeton Separations). The wild-type *attP* and *attB* site probes were further purified on 8% polyacrylamide (29:1). Mutant sites were used without further purification. Binding reactions contained 20 mM TrisHCl, pH 7.4, 150 mM KCl, 2 mM DTT, 25 µg/ml BSA, 25 µg/ml salmon sperm DNA, 5% glycerol, ~1 nM DNA, and varying amounts of integrase and were incubated for 30 min at 30° prior to electrophoresis on 6% polyacrylamide (37.5:1) using Tris-glycine buffer, pH 8.3, at 15°C. Dried gels were analyzed using a Storm phosphorimager (GE).

Intramolecular recombination in *E. coli*. Construction of the F' reporter followed that described by Gibb et al. (20) for Cre-loxP recombination. Briefly, two attachment sites (*attP*/*attB*, *attL*/*attR*, *attP*/*attP*, and *attB*/*attB*) were subcloned in the same orientation with respect to the crossover dinucleotide into pBluescriptIISK+ (Stratagene) so that they flank a 867 bp segment containing the transcriptional terminator from *rpoC*. This cassette was then subcloned into a FW11 (54) derivative containing a modified sulA promoter (55) and the *aadA* coding sequence. The resultant pFW11 was transformed into CSH100 cells and double crossovers onto the F' were selected following conjugation with CSH142 containing the heat-curable plasmid pTSA29 and identification of kanamycin/ampicillin resistant clones where pFW11 had lost the chloramphenicol marker. The F'-reporter is shown schematically in Fig. 5C. Excision of the terminator by the integrase results in streptomycin resistance and a *lac*+ phenotype.

pACYCBad1 integrase expression constructs were transformed into CSH142 cells containing the excision reporter and incubated for one hour at 37°C in SOC containing 0.2% arabinose. Although recombination occurred at high frequency in the absence of added arabinose, a small amount of arabinose was found to increase activity. The combination of 20 mM glucose from the SOC (which prevents induction) and 0.2% arabinose gave results similar to low amounts of added arabinose in the absence of glucose. Full induction with 0.2% arabinose in the absence of glucose inhibited recombination. Equal volumes were plated on LB containing chloramphenicol (35 µg/ml) and on LB with chloramphenicol (35 µg/ml) and streptomycin (20 µg/ml) and grown overnight at 37 °C. Activity was scored as the percentage of transformants that were resistant to streptomycin. In separate experiments using either Cre recombinase or LI integrase, we determined that a minimum incubation time of 45 min is required for expression of the streptomycin resistance marker. Thus, excision must occur within the first 15 min of incubation in order to contribute to the observed activity. In control experiments, transformation of the pACYCBad1-S10A catalytically defective mutant

typically resulted in 0-2 streptomycin-resistant colonies, compared to ~3000 streptomycin-resistant colonies observed when the pACYCBad1-WT integrase was transformed. All streptomycin-resistant colonies were also blue on plates containing X-gal. Individual excision product F' regions were sequenced for all four reporters to verify that the expected site-specific recombination event had occurred. All assays were performed in triplicate .

Intermolecular recombination in *E. coli*. To generate single copy *attP* and *attB* sites, we cloned BamHI-HindIII fragments containing 56-bp attachment sites into the same sites of pFW11 and generated F'-episomes in CSH142 as described above for the intra-molecular assay. pACYCBad1 constructs expressing WT integrase, S10A integrase, or ΔCC integrase were transformed into CSH142-F'-*attP* and -*attB* strains to generate integration reporters.

R6kγ suicide plasmids containing an attachment site or an att-site mutant were transformed into the appropriate reporter strain and incubated for 1 h at 37 °C in SOC + 0.2% arabinose. Transformations were plated on LB containing ampicillin (100 µg/ml) and grown overnight at 37 °C. Only cells where the R6ky plasmid has been integrated into the F' episome via recombination between the two *att* sites produce ampicillin resistant colonies. Assays were performed in triplicate and activity was scored as a percentage of *attP* integration into the *attB*-containing F' episome by the WT integrase. Transformation of pFBR6kamp lacking an attachment site typically resulted in 0-2 colonies compared to ~1200 colonies for *attP* x *attB* integration by WT integrase.

Construction of int-*attP*, int-*attB*, and int-*attL* models. The high degree of structural similarity between the LI integrase-*attP* half-site structure and the γδ-resolvase-site I structure (24) in the region close to the crossover site suggested that the resolvase/DNA complex (Fig. S2) would serve as a good template for connecting two LI int-*attP* half-sites together to form a full int-*attP* complex. The complete conservation of secondary structual elements observed between the TP901 integrase catalytic domain and the resolvase/invertase structures (31) further argues that the γδ-resolvase catalytic domain dimer would serve as an adequate model for the LI integrase catalytic domain for the purposes of considering overall domain architecture. To generate the LI int-*attP* model, base-pairs 1-5 of the LI CTD-*attP* half-site structure were superimposed onto the corresponding five base-pairs flanking the crossover TA dinucleotide in the resolvase-DNA complex. The resulting transformation was then applied to the entire CTD-*attP* half-site complex. A second CTD-*attP* half-site complex was then superimposed onto the second DNA half-site of the resolvase/DNA complex using the same procedure. The resolvase catalytic domains (residues 1-123) were retained and combined with LI integrase (residues 133-452) to provide a model for the intact integrase dimer. Aside from the introduction of phosphodiester linkages at the center of the *attP* site, no flexible modeling or energy minimization was performed. The resulting int-*attP* complex model deviates slightly from twofold symmetry, due to the asymmetry present in the resolvase/DNA template.

To construct a model of the LI int-*attB* complex, a transformation was calculated which superimposes base pairs 13-23 onto base pairs 8-18 within the left half-site of the int-*attP* complex. This transformation was then applied to the ZD bound to this half-site (residues 264-452) to shift it 5-bp. The RD-ZD linker was then re-connected by joining residues 263 and 264 and the linker geometry was energy minimized within COOT (residues 257-264). We did not attempt to model the linker in any other way, although it seems likely that these residues will interact with the minor groove of *attB*, but running in the opposite direction

relative to that observed in the CTD-*attP* structure. The same procedure was applied to the right half-site of the int-*attP* complex to generate the int-*attB* model. To construct a model of the LI int-*attL* complex, the right half-site of the int-*attP* complex was changed to an int-*attB* half-site using the procedure described above. The resulting model has an int-*attP* left half-site and an int-*attB* right half-site. We chose the best defined CC motif (CC1) to include in the *attP* and *attB* sites, but used the CC2 conformation for *attL* because it allows clearer visualization of the structural plausibility of intramolecular interactions between CC motifs on that site.

Construction of synaptic complex models. To generate models of LI integrase synaptic complexes, we superimposed CTD-*attP* and CTD-*attB* half-site complexes onto the half-sites of the tetrameric γδ-resolvase-DNA synaptic complex (pdb code 1ZR4). The superpositions were calculated using DNA C' atoms from base-pairs 4-13 of *attP* and *attB* (numbering as in Fig. 4) and the corresponding base-pairs in the cleaved *res* site I DNA, starting four base-pairs from the crossover site. The final model contains the resolvase NTD tetramer (residues 2-128) and four LI integrase CTD-half-site complexes.

We also constructed models of synaptic complexes by docking the required int-att site complexes together as rigid bodies to obtain the DNA synaptic complex geometry established for Tn3 resolvase using SAXS and SANS data (39). Briefly, the two int-att complexes were transformed into a coordinate system where **z** is along the dyad of the catalytic domains, **x** is initially parallel to a line connecting the centers of the two DNA half-sites, **y** was set to be orthogonal to **x/z** and **x** was reset to be orthogonal to **y/z**. The origin was defined at an arbitrary point along the catalytic domain dyad. The second int-att site complex was rotated 180° about **y** and translated along **z** to give a distance d = 62 Å as defined by Nollmann et al. (2004). The second int-att site complex was then rotated about **z** to give φ=20°, which is defined as the angle between **x** directions calculated for the two complexes (Nollmann et al.; 2004). The φ angle is essentially the angle between att sites when viewed down the **z** (dyad) axis. We did not adjust the rotation of the catalytic domains relative to the int-bound att site DNA. This was determined to be a small adjustment of σ=8° for the Tn3 resolvase synaptic complex (39). The same procedure was applied to generate *attP* x *attB*, *attP* x *attP*, *attB* x *attB*, and *attL* x *attR* complexes, where *attL* x *attR* is identical in overall architecture to an antiparallel *attL* x *attL* model. Complexes were generated with both positive and negative crossing angle signs and both led to the same conclusions for *attP x attB, attP x attP* and *attL x attR* synapsis as described in the text for the γδ-resolvase-based models. The *attB x attB* complex shows significantly more steric clashes between CC motifs in the γδ-resolvase-based model (which has a negative crossing angle) than was observed for a positive crossing angle using the Tn3 resolvase template.

Plasmids generated in this study.

Plasmid	Origin	Marker(s)	Parent	Purpose
pYP156	pMB1	kan	pET29b	Expression/purification of WT int
pYP166	pMB1	amp	pETDuet	Expression/purification of in CTD (133-452-LEH$_6$)
pGV2570	pMB1	kan	pET29b	Expression/purification of int ΔCC (1-341, 417-452)
pGV2634	pMB1	kan	pET29b	Expression/purification of int ΔZD (1-264)
pGV2674	pMB1	kan	pET29b	Expression/purification of int S10A catalytic mutant
pGV1364	p15A	chlor	pACYCBad1	Expression of WT int
pGV2573	p15A	chlor	pACYCBad1	Expression of int ΔCC (1-341, 417-452)
pGV2574	p15A	chlor	pACYCBad1	Expression of int S10A
pGV2593	p15A	chlor	pACYCBad1	Expression of int ΔZD (1-264)
pGV1901	p15A	kan/chlor	pFW11	attL-attR excision; for F' recombination
pGV1902	p15A	kan/chlor	pFW11	attP-attB excision; for F' recombination
pGV2588	p15A	kan/chlor	pFW11	attP-attP excision; for F' recombination
pGV2589	p15A	kan/chlor	pFW11	attB-attB excision; for F' recombination
pKR2572	F	kan	CSH100-F'	F' reporter for attP-attB excision
pGV2596	F	kan	CSH100-F'	F' reporter for attL-attR excision
pGV2597	F	kan	CSH100-F'	F' reporter for attP-attP excision
pGV2598	F	kan	CSH100-F'	F' reporter for attB-attB excision
pGV2341	p15A	kan/chlor	pFW11	attP site; for F' recombination
pGV2342	p15A	kan/chlor	pFW11	attB site; for F' recombination
pGV2392	F	kan	CSH100-F'	F' containing attP site
pGV2393	F	kan	CSH100-F'	F' containing attB site
pGV2345	R6kγ	amp	pFBR6kamp	suicide plasmid containing attP site
pGV2346	R6kγ	amp	pFBR6kamp	suicide plasmid containing attB site

Notes

1. attP and attB mutants for DNA-binding and integration experiments were made using pGV2345 and pGV2346 templates, respectively.

References

48. Otwinowski,Z. and Minor,W. (1997) Processing of X-ray diffraction data collected in oscillation mode. *Process. X-Ray Diffr. Data Collect. Oscil. Mode*, **276**, 307–326.

49. Liu,Q., Zhang,Z. and Hendrickson,W.A. (2011) Multi-crystal anomalous diffraction for low-resolution macromolecular phasing. *Acta Crystallogr Biol Crystallogr*, **67**, 45–59.

50. Terwilliger,T.C. and Berendzen,J. (1999) Automated MAD and MIR structure solution. *Acta Crystallogr Biol Crystallogr*, **55**, 849–61.

51. Cowtan,K. and Main,P. (1998) Miscellaneous algorithms for density modification. *Acta Crystallogr Biol Crystallogr*, **54**, 487–93.

52. Emsley,P. and Cowtan,K. (2004) Coot: model-building tools for molecular graphics. *Acta Crystallogr Biol Crystallogr*, **60**, 2126–32.

53. Brünger,A.T., Adams,P.D., Clore,G.M., DeLano,W.L., Gros,P., Grosse-Kunstleve,R.W., Jiang,J.S., Kuszewski,J., Nilges,M., Pannu,N.S., et al. (1998) Crystallography & NMR system: A new software suite for macromolecular structure determination. *Acta Crystallogr Biol Crystallogr*, **54**, 905–21.

54. Whipple,F. (1998) Genetic analysis of prokaryotic and eukaryotic DNA-binding proteins in Escherichia coli. *Nucleic Acids Res*, **26**, 3700–6.

55. Dmitrova,M., Younès-Cauet,G., Oertel-Buchheit,P., Porte,D., Schnarr,M. and Granger-Schnarr,M. (1998) A new LexA-based genetic system for monitoring and analyzing protein heterodimerization in Escherichia coli. *Mol Gen Genet*, **257**, 205–12.

56. Matsuura,M., Noguchi,T., Yamaguchi,D., Aida,T., Asayama,M., Takahashi,H. and Shirai,M. (1996) The sre gene (ORF469) encodes a site-specific recombinase responsible for integration of the R4 phage genome. *J Bacteriol*, **178**, 3374–6.

57. Breüner,A., Brøndsted,L. and Hammer,K. (2001) Resolvase-like recombination performed by the TP901-1 integrase. *Microbiology*, **147**, 2051–63.
